# Vocal taking turns is premature at birth and improved by the postnatal phonetic environment in marmosets

**DOI:** 10.1093/nsr/nwaf162

**Published:** 2025-04-24

**Authors:** Runze Qi, Yingxu Lin, Shanshan Liu, Xinyuan Cao, Min Xie, Chencen Yu, Hao Sun, Lixia Gao, Xinjian Li

**Affiliations:** Department of Neurology of the Second Affiliated Hospital and Interdisciplinary Institute of Neuroscience and Technology, Zhejiang University School of Medicine, Hangzhou 310020, China; Nanhu Brain-computer Interface Institute, Hangzhou 311100, China; NHC and CAMS Key Laboratory of Medical Neurobiology, MOE Frontier Science Center for Brain Science and Brain-machine Integration, School of Brain Science and Brain Medicine, Zhejiang University, Hangzhou 310058, China; Department of Neurology of the Second Affiliated Hospital and Interdisciplinary Institute of Neuroscience and Technology, Zhejiang University School of Medicine, Hangzhou 310020, China; Key Laboratory of Biomedical Engineering of the Ministry of Education, College of Biomedical Engineering and Instrument Science, Zhejiang University, Hangzhou 310027, China; Department of Neurology of the Second Affiliated Hospital and Interdisciplinary Institute of Neuroscience and Technology, Zhejiang University School of Medicine, Hangzhou 310020, China; Key Laboratory of Biomedical Engineering of the Ministry of Education, College of Biomedical Engineering and Instrument Science, Zhejiang University, Hangzhou 310027, China; Department of Neurology of the Second Affiliated Hospital and Interdisciplinary Institute of Neuroscience and Technology, Zhejiang University School of Medicine, Hangzhou 310020, China; Department of Neurology of the Second Affiliated Hospital and Interdisciplinary Institute of Neuroscience and Technology, Zhejiang University School of Medicine, Hangzhou 310020, China; Department of Neurology of the Second Affiliated Hospital and Interdisciplinary Institute of Neuroscience and Technology, Zhejiang University School of Medicine, Hangzhou 310020, China; Department of Neurology of the Second Affiliated Hospital and Interdisciplinary Institute of Neuroscience and Technology, Zhejiang University School of Medicine, Hangzhou 310020, China; Key Laboratory of Biomedical Engineering of the Ministry of Education, College of Biomedical Engineering and Instrument Science, Zhejiang University, Hangzhou 310027, China; Department of Neurology of the Second Affiliated Hospital and Interdisciplinary Institute of Neuroscience and Technology, Zhejiang University School of Medicine, Hangzhou 310020, China; Nanhu Brain-computer Interface Institute, Hangzhou 311100, China; NHC and CAMS Key Laboratory of Medical Neurobiology, MOE Frontier Science Center for Brain Science and Brain-machine Integration, School of Brain Science and Brain Medicine, Zhejiang University, Hangzhou 310058, China; Key Laboratory of Biomedical Engineering of the Ministry of Education, College of Biomedical Engineering and Instrument Science, Zhejiang University, Hangzhou 310027, China; Department of Neurology of the Second Affiliated Hospital and Interdisciplinary Institute of Neuroscience and Technology, Zhejiang University School of Medicine, Hangzhou 310020, China; Nanhu Brain-computer Interface Institute, Hangzhou 311100, China; NHC and CAMS Key Laboratory of Medical Neurobiology, MOE Frontier Science Center for Brain Science and Brain-machine Integration, School of Brain Science and Brain Medicine, Zhejiang University, Hangzhou 310058, China

**Keywords:** marmoset, non-human primate, vocalization, vocal communication, vocal development, antiphony, taking turns

## Abstract

Precisely time-controlled vocal antiphony is crucial for the social communication of arboreal marmosets. However, it remains unclear when this antiphony is formed and how postnatal acoustic environments affect its development. In the present study, we systematically recorded the emitted calls of infant marmosets in an antiphonal calling scenario from postnatal day one (P1) to postnatal 10 weeks (W10). We found that infant marmosets emit most types of adult calls and engage in turn-taking as early as in P1. In addition, parent-reared infants emitted more antiphonal phee calls than hand-reared marmosets in W10. Call transitions in parent-reared W10 animals mainly occurred between phee calls or from phee calls to other call types. In contrast, P1 and hand-reared W10 marmosets displayed call transitions among various types of calls. These findings suggest that the antiphony in marmosets emerges on P1 but remains immature, and the antiphony skills can be improved by development environments, especially by the vocal feedback from parents.

## INTRODUCTION

The phylogenetic origins of human speech have long been a hot topic in neuroscience [1–3]. The intriguing possibility of identifying precursors to speech in the vocalizations of non-human primates and other animal models remains challenging and is a subject of vivid discussion [4–8]. It is well known that most newborn animals, such as rodents, birds, bats and non-human primates, can emit a variety of calls just after birth [3,9,10], indicating the innate aspect of vocal production. However, the mechanism of vocal communication observed in several animal models [11,12] remains controversial. So far, several studies have investigated call production, and whether and how an abnormal postnatal phonetic environment, such as lacking parental vocal feedback, impairs the learning and development of vocal communication in non-human primates [13,14]. The traditional view holds that vocal production of non-human primates is largely innate [1,15] because infants that are born deaf or those raised in sound-isolated environments could produce all repertoire of conspecific calls [15,16]. However, Takahashi *et al.* recorded marmoset vocalizations on postnatal day one (P1) and they observed six types of calls, including a low proportion of phee calls, in newborn marmosets. They claimed that a subset of infant-only marmoset calls transformed into adult phee calls during development [4]. So, whether the vocal production of the marmoset is innate or learned remains controversial. In other words, which aspects of vocal development are genetically determined, and which parts are affected by sound environments?

Taking turns is a remarkable feature of human speech and crucial for social communication [12,17]. Human ‘turn-taking’ (antiphony) requires the participants to speak alternately and precisely control the initiation of the sentence to avoid talking simultaneously [17,18]. In addition, this skill is acquired through social interactions during individuals’ development [19–21]. An abnormal postnatal phonetic environment, especially lacking parental vocal feedback, impairs the development of human speech [22]. Many primates use turn-taking during vocal communication, termed antiphonal calling [23–29]. The phee is regarded as the primary communicational call type during antiphonal calling and is prevalent in juvenile and adult marmosets in both the wild and the laboratory environment [11,14,23,24,30]. In the past decade, several studies have shown that marmoset vocalizations, especially the phee call, are shaped by parental vocal feedback during early development [13,14,22,31,32], suggesting that social feedback modulates the vocal development of non-human primates. However, it remains unclear when taking turns in vocal communication is formed and how parental vocal feedback modulates the development of antiphonal calling in marmosets.

Here, by recording the vocalizations of infant marmosets that were raised in different phonetic environments across various developmental stages, we addressed (i) when the different types of calls were produced, (ii) whether the antiphonal calling exists in P1 and (iii) how the hand-reared environment, which lacks parental vocal feedback and replaces parent maternity with human maternity, affects the antiphonal calling of infant marmosets. These findings will elucidate the mechanisms underlying vocal ontogeny and the development of vocal communication in marmosets, which will shed light on the development of human language.

## RESULTS

### Predetermined vocalizations in newborn marmosets

**Figure 1.**
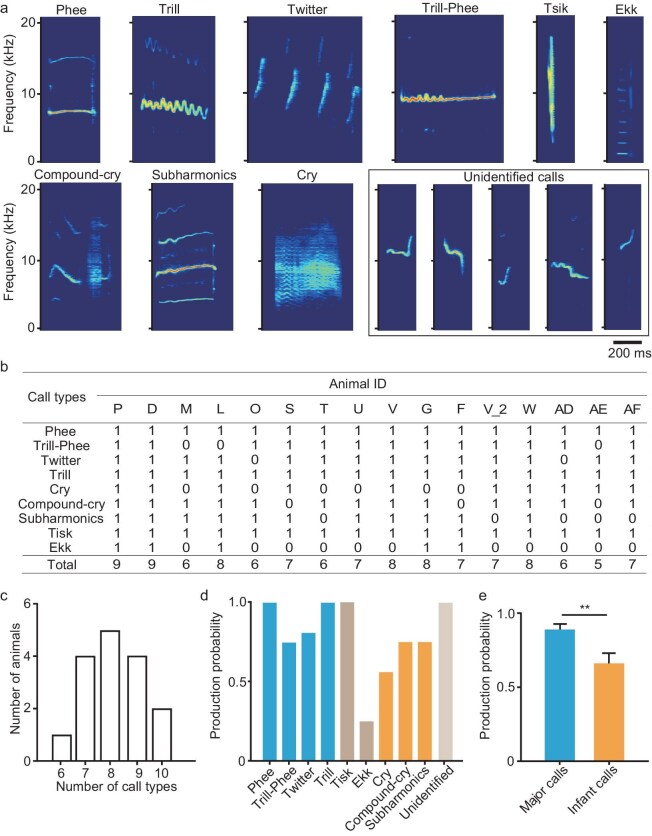
P1 marmosets emit the most repertoire of marmoset calls. (a) Examples showing different call types recorded from P1 marmosets. The call types are phee, trill-phee, twitter, trill, tsik, ekk, cry, compound-cry and subharmonic, as well as unidentified calls. (b) Table showing the call types emitted by individual P1 marmosets. The first column lists the call types. 1, recorded; 0, not recorded; P, D, M, L, O, S, T, U, V, G, F, V_2, W, AD, AE and AF are the animal identifications (Animal ID). (c) Distribution of call-type numbers produced by 16 P1 marmosets in a 5-min recording. (d) Probability of call type produced by P1 marmosets (*n* = 16). (e) Probability of the major calls (phee, trill-phee, twitter and trill) and infant calls (cry, compound-cry and subharmonic) produced by P1 marmosets (*n* = 16).

To dissect whether newborn marmosets can emit the entire repertoire of marmoset calls and whether these calls contribute to social communication, we transferred individual P1 marmosets together with their parents into a sound-proof room. We then recorded their calls for two sessions. The first session lasted for 2 min and the baby marmosets could not visually contact their parents, and the second session lasted for 3 min and the baby marmosets could visually contact their parents. We then visually recognized the different types of calls produced by the P1 marmosets and calculated the mean and standard deviation of major acoustic parameters of these visually identified calls (Table 1). Lastly, we fitted these major acoustic parameters into a normal distribution and used the 95th percentile as the threshold to classify identifiable and unidentifiable marmoset calls (Fig. S1). Based on these measurements, we further defined the major call types of P1 marmosets by using their acoustic parameters. For example, phee is 0.24–0.70 s in duration and is typically uttered with a dominant frequency of <9.9 kHz and a bandwidth of <3.4 kHz. We visually identified 2047 phee calls in P1 marmosets, 88% of which fell in the threshold and were classified as phee calls (Fig. S2a and b). Others (12%) were classified as phee-like calls (Fig. S2a and b). With this criterion, we identified nine types of calls at P1: phee, trill-phee, twitter, trill, tsik, ekk, cry, compound-cry and subharmonic (Fig. 1a and b). Statistically, individual P1 marmosets produced more than six types of calls within two recording sessions (Fig. 1b and c). Phee was previously regarded as a mature call that was transformed from infant-specific calls (cry, compound-cry and subharmonic) [4]. However, we found that all the P1 marmosets emitted phee calls (Fig. 1b and d). In contrast, only 10 out of 16 (62.5%) animals produced cry calls (Fig. 1b and d). In addition, P1 marmosets had a higher probability of producing the major adult calls (phee, trill-phee, trill and twitter) rather than infant-specific calls (Fig. 1d and e; 1e, *P* = 0.003, Wilcoxon test). These results demonstrated that newborn marmosets have largely innate vocal production even at P1.

**Table 1.** Acoustic features of P1 marmosets in an antiphonal calling scenario.

	Call types					
	Phee	Trill	Trill-phee	Cry	Tsik	Twitter
Acoustic features	(*n* = 1795)	(*n* = 357)	(*n* = 21)	(*n* = 318)	(*n* = 120)	(*n* = 292)
Duration (s)	0.36 ± 0.06	0.25 ± 0.08	0.34 ± 0.10	0.36 ± 0.13	0.07 ± 0.02	0.61 ± 0.31
Bandwidth (kHz)	1.94 ± 0.58	3.24 ± 1.15	1.82 ± 0.52	8.09 ± 1.71	13.76 ± 3.18	6.89 ± 1.51
Center frequency (kHz)	8.26 ± 0.74	9.70 ± 0.80	8.96 ± 0.71	8.28 ± 0.85	8.50 ± 1.39	11.03 ± 0.89
Dominant frequency (kHz)	8.23 ± 0.76	9.67 ± 0.89	9.02 ± 0.82	8.10 ± 1.27	8.18 ± 1.67	11.13 ± 1.24
Minimal frequency (kHz)	7.44 ± 0.69	8.02 ± 1.15	8.12 ± 0.75	4.62 ± 0.77	4.01 ± 1.05	14.47 ± 0.91
Maximal frequency (kHz)	9.38 ± 0.79	11.26 ± 1.14	9.95 ± 0.62	12.7 ± 1.56	17.78 ± 2.50	7.58 ± 1.36

Duration: length of the vocalization; bandwidth: frequency bandwidth across a call; central frequency: frequency with the maximal energy within a call; dominant frequency: frequency corresponding to the maximum in the spectrum; minimal frequency: minimum frequency of fundamental frequency; maximum frequency: maximum frequency of fundamental frequency. The data indicate mean ± standard deviation.

### Antiphonal interactions occurred in P1 marmosets

**Figure 2.**
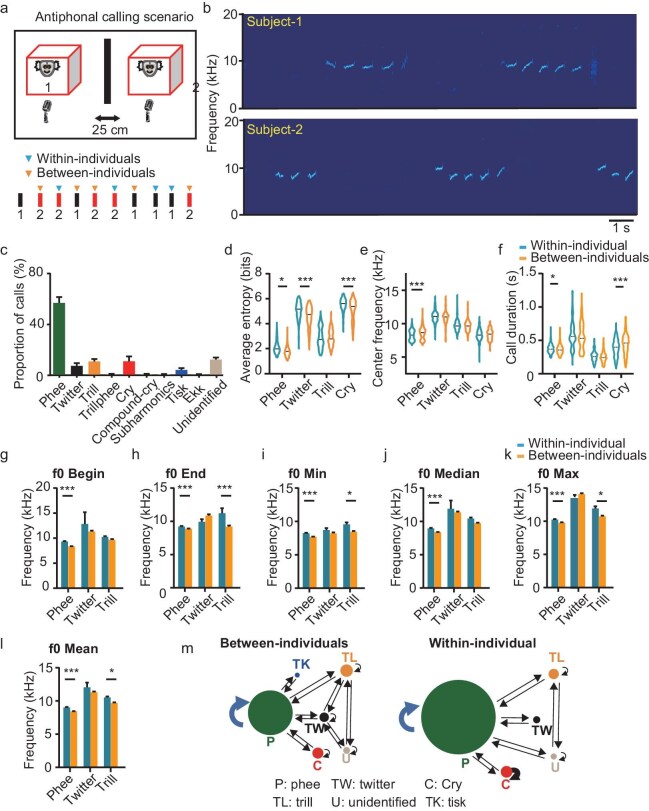
Comparison of acoustical parameters between ‘within-individual’ and ‘between-individuals’ calls produced by P1 marmosets in the antiphonal calling scenario. (a) Upper panel: experimental set-up for antiphonal calling. Two marmosets are separated by a black cloth curtain halfway between the two animals, which are 25 cm apart. Lower panel: schematic image showing the classification of ‘within-individual’ calls (blue arrows) and ‘between-individuals’ calls (orange arrows). Black bars represent phee calls from Marmoset 1; red bars represent phee calls from Marmoset 2. (b) Spectrograms of the vocal sequences produced by two marmosets in an antiphonal calling scenario. Upper panel: calls from Subject 1; lower panel: calls from Subject 2. (c) Proportion of different types of calls emitted by P1 marmosets in the antiphonal calling scenario (*n* = 8 pairs). (d–f) Comparison of (d) averaged entropy, (e) center frequency and (f) duration of ‘between-individuals’ and ‘within-individual’ calls produced by the same animals. (g–l) Comparison of different parameters of fundamental frequency (f0) between ‘between-individuals’ and ‘within-individual’ calls produced by the same P1 marmosets. (g) Fundamental frequency of call begins (f0 Begin); (h) fundamental frequency of call ends (f0 End); (i) minimal fundamental frequency (f0 Min); (j) median fundamental frequency (f0 Median); (k) maximal of the fundamental frequency (f0 Max); (l) averaged fundamental frequency (f0 Mean). (d)–(l) *P < 0.05; **P < 0.01; ***P < 0.001. (m) Transition diagram showing the call sequences of ‘between-individuals’ calls (left) and ‘within-individual’ calls (right) of eight pairs of P1 marmosets in an antiphonal calling scenario. Each node corresponds to a type of call and the arrows correspond to the transitions between call types. The size of a node is the proportion of call types and the thickness of an arrow represents the transition probability. P1 marmosets can switch their calls to any call type, although most transitions occur between phee calls. P, phee (green); TW, twitter (black); C, cry (red); TL, trill (orange); Tk, tsik (blue); U, unidentified (gray).

Although P1 marmosets emit the most repertoire of adult calls, it remains unclear whether antiphonal interactions occur in P1 marmosets and which call types are used for antiphonal interaction. To address these questions, we examined the turn-taking during vocal communication by using 8 pairs of 10 P1 marmosets (2 twins and 2 triplets; Table 2) in the antiphonal calling scenario, which is similar to the experimental setting of adult animals in previous studies [24,33]. Two siblings were separated into two visually occluded boxes and their calls were recorded for 5 min by using two microphones positioned close to each animal (Fig. 2a, upper panel). Based on the intensity difference (∼15 dBFS) of each call recorded by the two microphones, we identified the caller for each call (Fig. 2b; see details in the Methods section). P1 marmosets mainly emitted phee calls when visually occluded and separated from their conspecifics (Fig. 2c), consistently with the previous finding that adult marmosets would emit more phee calls under this condition [33,34].

**Table 2.** Vocal activity of P1 marmosets in an antiphonal calling scenario.

Recording pairs	Animal ID	Total call number	Calling rate (calls/min)	Phee rate (calls/min)	Recording duration (s)
U-V1	U	269	42.47	16.26	380
	V1	316	49.89	26.05	
T-V1	T	106	19.69	11.70	323
	V1	299	55.54	29.16	
T-U	T	223	42.34	26.20	316
	U	288	54.68	26.39	
O-P	O	82	14.86	13.96	331
	P	124	22.48	18.13	
W-V2	V2	297	59.60	28.90	299
	W	304	61.00	47.36	
AD-AE	AE	128	25.51	24.52	301
	AD	245	48.84	35.88	
AE-AF	AF	248	49.44	21.93	301
	AE	246	49.04	32.09	
AD-AF	AF	261	52.37	20.07	299
	AD	187	37.53	25.48	
Mean		226.4	42.8	25.3	318
Sum		3623	—	—	2550

Animals T, U and V1 are triplets; AD, AE and AF are triplets; O and P are twins; W and V2 are twins.

To examine whether the two infant marmosets had vocal interactions, we categorized the calls produced by the same animal into ‘within-individual’ and ‘between-individuals’ calls (Fig. 2a, lower panel). A call was defined as ‘within-individual’ if the preceding call was produced by the same caller; otherwise, a call was defined as ‘between-individuals’ [24]. So, the ‘between-individuals’ call is correlated with vocal communication between two animals. We then compared the acoustic parameters between ‘within-individual’ and ‘between-individuals’ calls, including the inter-call interval (ICI, Fig. S2c–e), inter-onset interval (IOI, Fig. S2f), Wiener entropy (Fig. 2d), center frequency (Fig. 2e), call duration (Fig. 2f) and various parameters of the fundamental frequency (f0, Fig. 2g–l). We found that the ICI distribution of ‘within-individual’ calls ranged from 0 to 1 s whereas that of ‘between-individuals’ calls ranged from –1 to 1 s relative to the offset of the initial phee call (Fig. S2c and d). Statistically, the ICI and IOI of ‘within-individual’ calls were significantly longer than those of ‘between-individuals’ calls (Fig. S2e, Mann-Whitney *U* test, *P* < 0.001, Fig. S2f, Mann-Whitney *U* test, *P* < 0.001). The averaged Wiener entropy of ‘within-individual’ calls, including phee, twitter and cry, was higher than that of the same type of ‘between-individuals’ calls (Fig. 2d, two-way analysis of variance (ANOVA), F = 50.046, *P* < 0.001). The center frequency of ‘within-individual’ phee calls was lower than that of ‘between-individuals’ phee calls (Fig. 2e, two-way ANOVA, F = 12.426, *P* < 0.001). The duration of ‘within-individual’ phee calls was longer than that of ‘between-individuals’ phee calls whereas the duration of ‘within-individual’ cry calls was shorter than that of ‘between-individuals’ cry calls (Fig. 2f, two-way ANOVA, F = 13.643, *P* < 0.001). After the different acoustic parameters were normalized, the difference in the entropy of phee calls disappeared, while it remained significant for the other three call types (Fig. S2g, two-way ANOVA, F = 12.691, *P* < 0.001). Significant differences were still found in the duration and center frequency of phee calls (Fig. S2h, two-way ANOVA, F = 11.123, *P* < 0.001; Fig. S2i, two-way ANOVA, F = 17.685, *P* < 0.001). Moreover, we found significant frequency differences in various f0 parameters between ‘within-individual’ and ‘between-individuals’ phee and trill calls (Fig. 2g–l, two-way ANOVA; f0 Begin, F = 0.596, *P* = 0.551; f0 End, F = 6.866, *P* = 0.001; f0 Min, F = 1.152, *P* = 0.316; f0 Median, F = 0.071, *P* = 0.932; f0 Max, F = 1.031, *P* = 0.357; f0 Mean, F = 0.299, *P* = 0.741). Lastly, we quantified the call transitions of ‘between-individuals’ and ‘within-individual’ calls by using the previous methods [13,35]. We found that most call transitions occurred between phee calls regardless of ‘within-individual’ and ‘between-individuals’ calls (Fig. 2m). In addition, the call sequence of ‘between-individuals’ calls indicated that P1 marmosets could switch their calls between any call types (Fig. 2m, left) whereas the call sequence of ‘within-individual’ calls exhibited all transitions within phee calls or between phee and other calls (Fig. 2m, right).

**Figure 3.**
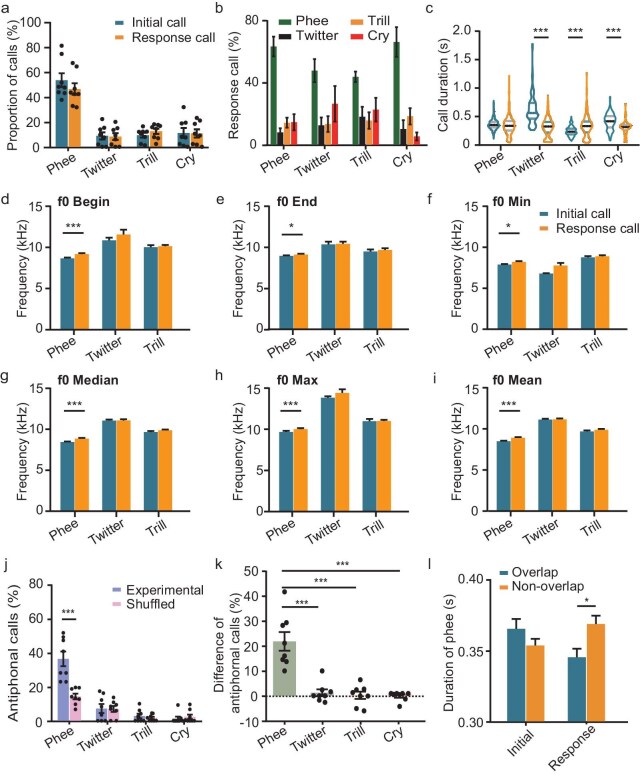
Antiphonal communication between P1 marmosets. (a) Proportions of phee, twitter, trill and cry emitted by P1 marmosets in the antiphonal calling scenario as the initial call or the response call (*n* = 8 pairs of marmosets). (b) Proportions of phee, twitter, trill and cry as a response call if the initial call was phee, twitter, trill and cry. *x*-axis, initial call types. (c) Averaged duration of phee, twitter, trill and cry as the initial call (blue) or the response calls (orange). (d–i) Comparison of different parameters of fundamental frequency (f0) between the initial call and the response calls of P1 marmosets. (d) f0 Begin; (e) f0 End; (f) f0 Min; (g) f0 Median; (h) f0 Max; (i) f0 Mean. (j) Proportions of phee-phee, twitter-twitter, trill-trill and cry-cry pairs emitted by P1 marmosets in the antiphonal calling scenario (*n* = 8 pairs), which were compared to those after the call sequences were randomly shuffled. (k) Proportional difference of antiphonal calls between experimental and shuffled datasets for phee-phee, twitter-twitter, trill-trill and cry-cry call pairs. Only the proportion of phee-phee pairs was higher than the chance level. (l) Comparison of phee durations between the initial or the response phee; and between the overlapped phee or the non-overlapped phee. (c)–(l), *P < 0.05; **P < 0.01; ***P < 0.001.

We then categorized each ‘between-individuals’ call pair into ‘initial calls’ and ‘response calls’ based on the temporal sequence of the calls produced by the two callers. We found similar proportions of initial and response calls for phee, twitter, trill and cry (Fig. 3a, two-way ANOVA, F = 1.421, *P* = 0.234). However, P1 marmosets responded mainly with phee calls, regardless of the initial call type (Fig. 3b, two-way ANOVA, F = 2.206, *P* = 0.027). We then analysed the acoustic parameters of ‘initial calls’ and ‘response calls’, including call duration and various parameters of f0. We found that the duration of phee calls as ‘initial calls’ did not differ significantly from that of phee calls as ‘response calls’. In contrast, the duration of other call types as ‘initial calls’ differed significantly from that of the ‘response calls’ (Fig. 3c, F = 135.458, *P* < 0.001). Regarding different parameters of f0, significant differences were found between ‘initial calls’ and ‘response calls’ for phee calls but not for other call types (Fig. 3d–i, two-way ANOVA, f0 Begin, F = 0.395, *P* = 0.674; f0 End, F = 0.644, *P* = 0.526; f0 Min, F = 0.767, *P* = 0.465; f0 Median, F = 1.087, *P* = 0.338; f0 Max, F = 0.091, *P* = 0.913; f0 Mean, F = 0.936, *P* = 0.393). So, P1 marmosets may modulate the acoustic parameters of their calls during vocal interactions with conspecifics.

To further confirm the existence of vocal interactions in P1 marmosets, we analysed the antiphonal communication of paired P1 marmosets by using the previous methods [24]. We found that ∼35% of ‘between-individuals’ phee calls met the criteria for antiphonal calling; this was much higher than for other call types (Fig. 3j, two-way ANOVA, F = 53.771, *P* < 0.001). Furthermore, after the call sequences of the two animals were randomized, the proportion of antiphonal phee calls decreased significantly (Fig. 3j, two-way ANOVA, F = 13.290, *P* < 0.001). Although some ‘between-individuals’ twitter, trill and cry calls also had intervals below the ICI threshold, their proportions were similar to those of the shuffled dataset (Fig. 3j). Taken together, ∼20% of ‘between-individuals’ phee calls (the difference between the experimental dataset and the shuffled dataset) were classified as antiphonal calls; this proportion was significantly higher than that of other call types and above the chance level (Fig. 3k, one-way ANOVA, F = 26.010, *P* < 0.001).

Next, we analysed the time cross-correlation of phee calls produced by the two infant marmosets. Two positive peaks were found at a time lag of ±4 s (Fig. S2j) and this time lag was mainly contributed to by the ‘within-individual’ calls (Fig. S2k). Only one positive peak was found at ∼0.2 s in the time cross-correlation of ‘between-individuals’ calls (Fig. S2l), which revealed that marmosets tended to respond to a call within 0–2 s following a call produced by another marmoset (Fig. S2l). Last, we analysed the ICI distribution between initial and response phee calls in P1 marmosets, which ranged from –0.6 to 1 s (Fig. S2m). Importantly, most ICIs were shorter than the antiphonal calling threshold (mean + std, red dashed line). However, when the call sequence was shuffled, the ICI distribution expanded from –0.7 to 3 s (Fig. S2m), with more calls over the ICI threshold. These results further revealed the existence of antiphonal calling in P1 marmosets. Furthermore, we found that a non-overlapped response phee had a longer duration than an overlapped response phee (Fig. 3l, two-way ANOVA, F = 4.808, *P* = 0.029), supporting the idea that P1 marmosets may modulate the spectrotemporal acoustic structures of their vocalizations during vocal interactions. Taken together, turn-taking in vocal interactions exists in newborn marmosets.

### Different call transitions in parent-reared and hand-reared infant marmosets

**Figure 4.**
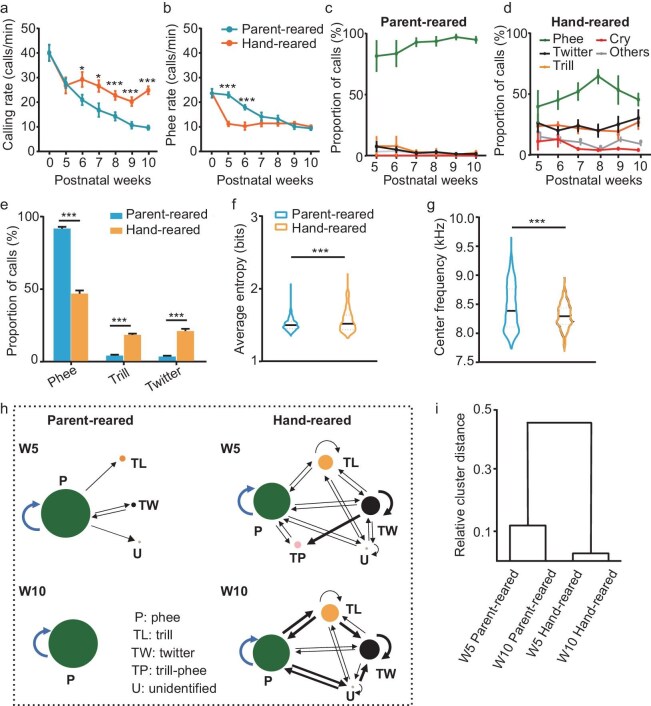
Distinct call usage and call transitions between the hand-reared and parent-reared groups in the antiphonal calling scenario. (a) Calling rate of all calls produced by the hand-reared (*n* = 4) and parent-reared (*n* = 4) marmosets in the antiphonal calling scenario from postnatal W0 to W10. (b) Calling rate of phee in the hand-reared and parent-reared marmosets from postnatal W0 to W10. (c and d) Proportions of different types of calls produced by the (c) parent-reared (*n* = 4) and (d) hand-reared (*n* = 4) marmosets in the antiphonal calling scenario across ages (W5–W10). (e) Proportions of phee, trill and twitter emitted by infant marmosets from the hand-reared (*n* = 4) and parent-reared (*n* = 4) groups in the antiphonal calling scenario at W10. (f) Average entropy and (g) center frequency of phee calls in hand-reared (*n* = 4) and parent-reared (*n* = 4) groups at W10. (a)–(g), *P < 0.05; **P < 0.01; ***P < 0.001. (h) Transition diagrams showing the call sequences of parent-reared (*n* = 4) and hand-reared (*n* = 4) groups at W5 and W10 in the antiphonal calling scenario. The color of each node corresponds to a type of call and the arrows correspond to the transitions between call types. The sizes of the nodes are the proportion of the call type and the thickness of an arrow represents the transition probability. P, phee (green); TW, twitter (black); TL, trill (orange); TP, trill-phee (pink); U, unidentified (gray). (i) Clustering dendrogram of infant marmosets in two groups (parent-reared, *n* = 4; hand-reared, *n* = 4) at two developmental ages (W5 and W10) based on call distribution and transitions.

Previous studies have shown that vocal feedback from parents plays a key role in the development of vocal production in infant marmosets [4,13,21,32]; however, it is unclear whether and how postnatal sound environments affect vocal communication, especially for the turn-taking conversations in the antiphonal calling scenario. To save as many marmoset babies as possible, we developed hand-rearing methods in which a subset of infant marmosets were fed by well-trained caretakers (see Methods) and parent maternal care was replaced by human maternity [36]. Importantly, although hand-reared marmosets could hear and communicate with hand-reared littermates, they had never heard adult marmoset vocalizations and lacked vocal interaction with their parents during the early vocal development period. Vocal recordings were then conducted for both hand-reared and parent-reared groups in the antiphonal calling scenario from postnatal Weeks 5 to 10 (W5–W10). We found that the body weights increased gradually in both groups (Fig. S3a, two-way ANOVA, F = 2.261, *P* = 0.257), similarly to the reports in previous studies [4,37]. Interestingly, the calling rate (Fig. 4a, two-way ANOVA, F = 26.782, *P* < 0.001) and the number of call types (Fig. S3b, two-way ANOVA, F = 10.342, *P* < 0.001) in the parent-reared group gradually decreased as the individuals developed, which is consistent with previous studies [4,38]. In contrast, the hand-reared group exhibited higher calling rates (Fig. 4a, two-way ANOVA, F = 29.761, *P* < 0.001) and more call types (Fig. S3b, two-way ANOVA, F = 6.309, *P* < 0.001). Moreover, the parent-reared group produced significantly more phee calls than the hand-reared group from W5 to W6 (Fig. 4b, two-way ANOVA, F = 7.613, *P* < 0.001). More specifically, animals in the parent-reared group emitted a higher proportion of phee calls and lower proportions of trill and twitter calls than the hand-reared group (Fig. 4c–e; two-way ANOVA, 4c, F = 1.600, *P* = 0.227; 4d, F = 2.503, *P* = 0.042; 4e, F = 101.651, *P* < 0.001). We then calculated the mean and coefficient of variation (CV) of major acoustic parameters of phee calls across different postnatal ages and groups, as shown in Table 3. We found that the CV of phee duration was higher in the hand-reared group than that in the parent-reared group. However, the CV of the dominant frequency in the hand-reared group is not different from that of the parent-reared group (Table 3). So, the phee calls that were produced by the hand-reared group were less stable in duration but more stable in dominant frequency. Next, we compared the average entropy and center frequency of phee calls in the two groups at W10, which displayed higher entropy and lower center frequency in the hand-reared group than in the parent-reared group (Fig. 4f and g; Wilcoxon test; averaged entropy, *P* < 0.001; center frequency, P < 0.001). We further compared the differences in entropy, dominant frequency, call duration and different parameters of f0 between the two groups (Fig. S3c–e, l–m). We found that the average entropy of phee in the parent-reared group was significantly lower than that of the hand-reared group across all development ages (Fig. S3c, two-way ANOVA, F = 7.178, P < 0.001). No significant difference was found in the dominant frequency of phee calls between the two groups (Fig. S3d, two-way ANOVA, F = 1.351, *P* = 0.284). The duration of phee calls produced by the parent-reared group gradually elongated as the animals grew older and became significantly longer than that of the hand-reared group on W9–W10 (Fig. S3e, two-way ANOVA, F = 2.276, *P* = 0.057). We also compared the ‘within-individual’ and ‘between-individuals’ phee calls of both parent-reared and hand-reared groups by normalizing the different acoustic parameters (Fig. S3f–k). We found that the normalized entropy decreased with animal development for both ‘within-individual’ and ‘between-individuals’ phee calls, but no significance difference was found between the two categories of calls (Fig. S3i, two-way ANOVA, F = 1.565, *P* = 0.114). However, significance differences were found at W5 and W10 between the parent-reared and hand-reared groups after the entropy was normalized (Fig. S3f, two-way ANOVA, F = 2.174, *P* = 0.041). Interestingly, the normalized dominant frequency of the parent-reared group increased with development whereas that of the hand-reared group decreased with development (Fig. S3g, two-way ANOVA, F = 9.366, *P* < 0.001), but no significant difference was found in the normalized dominant frequency between ‘within-individual’ and ‘between-individuals’ phee calls (Fig. S3j, two-way ANOVA, F = 1.431, *P* = 0.209). The normalized phee duration increased faster for the parent-reared animals than for the hand-reared animals, with the difference displayed at postnatal W5 and W9–W10 (Fig. S3h, two-way ANOVA, F = 7.732, *P* < 0.001). The normalized phee duration of ‘within-individual’ and ‘between-individuals’ phee calls increased in parallel (Fig. S3k, two-way ANOVA, F = 6.882, *P* < 0.001). The f0 Begin, f0 End, f0 Min, f0 Median and f0 Mean of phee calls at W5 (Fig. S3l, two-way ANOVA, F = 25.568, *P* < 0.001) and the f0 Begin, f0 Min, f0 Median and f0 Max of phee calls at W10 (Fig. S3m, two-way ANOVA, F = 85.741, *P* < 0.001) in the parent-reared group were significantly different from those in the hand-reared group. These results indicate that the infant marmosets that were raised by parents exhibited different spectrotemporal structures in their phee calls compared with the hand-reared animals.

**Table 3.** Mean and CV of phee duration and dominant frequency of the parent-reared and hand-reared groups at different developmental ages.

	Parent-reared group				Hand-reared group			
	Duration (s)		Dominant frequency (kHz)		Duration (s)		Dominant frequency (kHz)	
Ages	Mean	CV	Mean	CV	Mean	CV	Mean	CV
W0	0.33	0.16	9.06	0.08	0.35	0.17	8.02	0.09
W5	0.65	0.15	8.16	0.07	0.74	0.28	8.76	0.05
W6	0.73	0.20	8.36	0.05	0.78	0.24	8.62	0.04
W7	0.83	0.23	8.51	0.05	0.88	0.27	8.57	0.03
W8	0.92	0.17	8.69	0.04	0.92	0.20	8.50	0.04
W9	1.08	0.18	8.62	0.03	1.00	0.22	8.37	0.03
W10	1.11	0.16	8.55	0.03	1.07	0.23	8.17	0.03

CV = SD/mean.

Lastly, we analysed the call transitions of the parent-reared and the hand-reared groups at W5 and W10. Interestingly, the call transitions significantly differed between the parent-reared and hand-reared groups, especially at W10 (Fig. 4h). The calls of the parent-reared group at W5 transitioned between phees or from phee to other calls whereas those at W10 only transitioned between phee calls (Fig. 4h, left). In contrast, although the calls of the hand-reared group mainly transitioned between phee calls, they also switched between all types of calls at both W5 and W10 (Fig. 4h, right). These results were further validated by *post hoc* cluster analysis, which showed that the call sequences were significantly different between groups rather than ages (Fig. 4i). We also analysed the call transitions for both ‘within-individual’ and ‘between-individuals’ calls in parent-reared and hand-reared groups at W5 and W10 (Fig. S4). We found that call transitions of ‘between-individuals’ (Fig. S4a and b) were comparable to those of ‘within-individual’ (Fig. S4c and d) at both W5 (Fig. S4a and c) and W10 (Fig. S4b and d). The main differences were from the parent-reared and hand-reared groups rather than from ‘within-individual’ or ‘between-individuals’ calls, and ages (Fig. S4). In summary, hand-reared infant marmosets emitted various types of calls and displayed complex call transitions during the antiphonal calling scenario, likely remaining in the ‘babbling’ stage at W10 and suggesting a developmental delay in vocal communication compared with the parent-reared group.

### Improvement of antiphonal interactions by the postnatal phonetic environment

**Figure 5.**
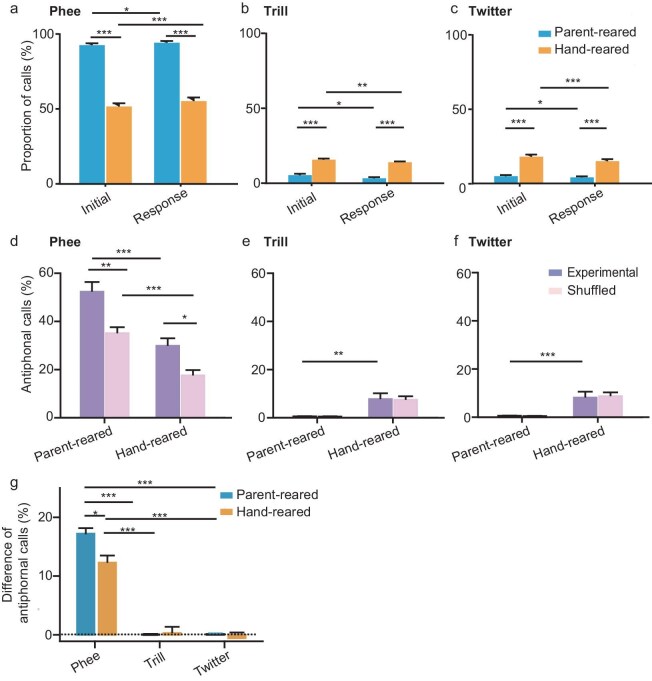
Antiphonal vocal interactions are improved by the postnatal phonetic environment. (a–c) Proportions of (a) phee, (b) trill and (c) twitter as the initial call and the response call from the parent-reared (*n* = 4) and hand-reared (*n* = 4) groups at W10 in the antiphonal calling scenario. (d–f) Proportions of (d) phee-phee, (e) twitter-twitter and (f) trill-trill pairs from the parent-reared (*n* = 4) and hand-reared (*n* = 4) groups at W10 compared with those of the shuffled datasets. (g) Proportion difference of phee-phee, trill-trill and twitter-twitter pairs between experimental and shuffled datasets for both parent-reared (*n* = 4) and hand-reared (*n* = 4) marmosets at W10. (a)–(g), *P < 0.05; **P < 0.01; ***P < 0.001.

The above results indicated that the postnatal phonetic environment affects vocal production and call transitions in the antiphonal calling scenario. However, it remains unclear whether parental vocal feedback affects vocal interactions, especially for taking turns. First, we separately compared the proportion of different call types as initial or response calls between the parent-reared and hand-reared groups (Fig. 5a–c). We found that the parent-reared group exhibited a higher percentage of phee calls and a lower percentage of trill and twitter calls, as both initial and response calls, compared with the hand-reared group (Fig. 5a–c; two-way ANOVA; 5a, F = 0.116, *P* = 0.734; 5b, F = 0.013, *P* = 0.911; 5c, F = 0.838, *P* = 0.362). We then compared the ICI and IOI at postnatal W5 and W10 for both groups (Fig. S5). The ICI distribution of ‘between-individuals’ phee calls in parent-reared groups has a broader width than that in the hand-reared group (Fig. S5a, b, e and f). In contrast, the ICI distribution of ‘within-individual’ phee calls in the parent-reared group was similar to that in the hand-reared group (Fig. S5a, b, e and f). Statistically, both the ICI and IOI of ‘within-individual’ calls were longer than those of ‘between-individuals’ calls in both the parent-reared group (Fig. S5c and d; two-way ANOVA; S5c, F = 302.282, *P* < 0.001; S5d, F = 109.712, *P* < 0.001) and the hand-reared group at W5 and W10 (Fig. S5g and h; two-way ANOVA; S5g, F = 414.521, *P* < 0.001; S5h, F = 382.712, *P* < 0.001). The ICI and IOI of both ‘within-individual’ and ‘between-individuals’ calls increased from W5 to W10 in the parent-reared group (Fig. S5c and d, two-way ANOVA; S5c, F = 162.041, *P* < 0.001; S5d, F = 82.352, *P* < 0.001) but not in the hand-reared group (Fig. S5g and h; two-way ANOVA; S5g, F = 1.461, *P* = 0.230; S5h, F = 3.321, *P =* 0.051).

Last, we compared the proportion of phee-phee, twitter-twitter and trill-trill calls as potential antiphonal call pairs between the parent-reared and hand-reared groups, as well as between experimental and shuffled datasets. We found that both the parent-reared and the hand-reared groups showed a significantly higher percentage of phee-phee antiphonal call pairs than the shuffled dataset (Fig. 5d, two-way ANOVA, F = 0.658, *P* = 0.421) but not for trill-trill call pairs (Fig. 5e, two-way ANOVA, F = 0.003, *P* = 0.953) and twitter-twitter call pairs (Fig. 5f, two-way ANOVA, F = 0.017, *P* = 0.898). More importantly, a higher percentage of phee-phee antiphonal calls was found in the parent-reared group than in the hand-reared group (Fig. 5g, two-way ANOVA, F = 1.415, *P* = 0.250). Therefore, antiphonal interactions were reinforced by the parental phonetic environment during the animal's development.

### The temporal overlaps between initial and response calls decrease during development

**Figure 6.**
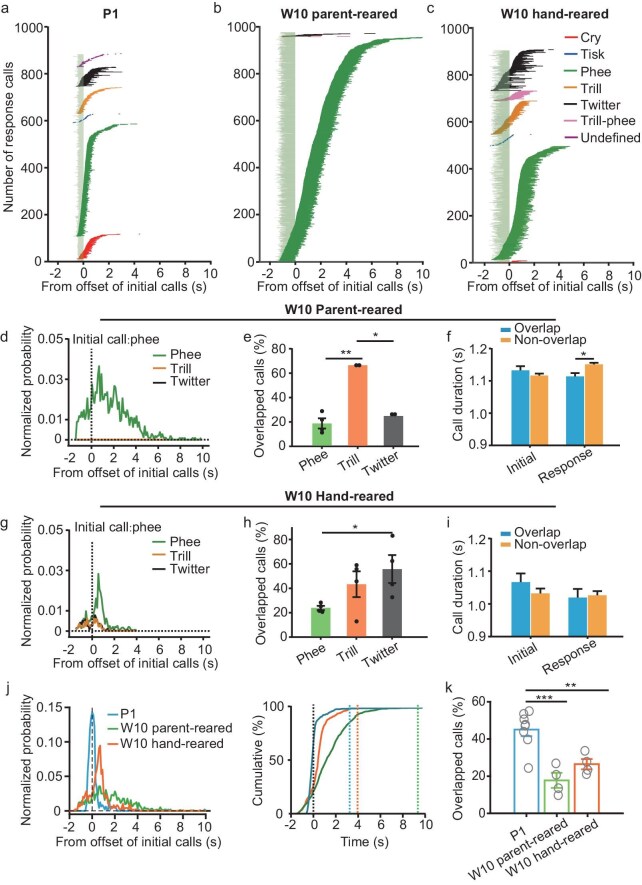
An increased gap of silence and decreased overlaps between initial and response calls during vocal development. (a–c) Example showing the temporal relationship between initial phee and various types of response calls at (a) P1, (b) W10 of the parent-reared group and (c) the hand-reared group. The initial phee calls and the response calls were aligned by the offset of the initial phee calls and sorted by the length of ICI for each type of response call. The colors in the response calls indicate different call types. (d, g) ICI distributions of initial phee call to various types of response calls (phee-phee, green; phee-trill, orange; phee-twitter, black) for W10 marmosets for the parent-reared group (d) and the hand-reared group (g). The distributions were normalized by the number of ‘phee-all’ call pairs. 0, the offset of the initial phee calls. (e, h) Proportions of overlapped phee-phee (green); phee-trill (orange); and phee-twitter (black) pairs in the parent-reared (e) and hand-reared (h) groups at W10. (f and i) Comparison of phee durations between the initial or the response phee; and between overlapped phee or no overlapped phee in the (f) parent-reared and (i) hand-reared groups at W10. (j) Normalized distributions (left) and Kolmogorov–Smirnov analysis (right) of ICI for phee-phee pairs among P1 (blue) and W10 of the parent-reared (green) and hand-reared groups (orange). black) for W10 marmosets for (d) the parent-reared group and (g) the hand-reared group. The distributions were normalized by the number of phee-phee call pairs. (k) Percentages of overlapped phee-phee pairs in the P1, hand-reared W10 and parent-reared W10 marmosets. *P < 0.05; **P < 0.01; ***P < 0.001.

To study the temporal relationship between initial and response calls, we selected call pairs consisting of an initial phee followed by various response call types from the P1, W10 parent-reared and W10 hand-reared groups. We then aligned the calls to the offset of the initial phee and the sequences of the call pairs were sorted by the length of ICI (Fig. 6a–c). Interestingly, parent-reared animals at W10 mainly emitted phee calls as responses within 1–4 s (Fig. 6b). On average, ∼25% of response calls (phee, trill and twitter) overlapped with the initial phee (Fig. 6b and d). In contrast, similarly to P1 (Fig. 6a), animals in the hand-reared group emitted diverse call types in response to phee calls (Fig. 6c). Also, ∼33% of response calls overlapped with the initial phee (Fig. 6c and g), especially with a higher proportion of overlapped phee-twitter and phee-trill calls (Fig. 6h). The proportion of overlapped phee-phee call pairs was the smallest compared with the other call pairs (phee-trill and phee-twitter) in both the parent-reared group (Fig. 6e, one-way ANOVA, F = 38.394, *P* = 0.001) and the hand-reared group (Fig. 6h, one-way ANOVA, F = 3.153, *P* = 0.092). Interestingly, the duration of overlapped response phee calls was significantly shorter than that of non-overlapped ones in the parent-reared group at W10 (Fig. 6f, two-way ANOVA, F = 8.416, *P* = 0.004); in contrast, no significant difference was found in the duration of response phee calls in the hand-reared group at the same age (Fig. 6i, two-way ANOVA, F = 0.996, *P* = 0.319). Next, we analysed the temporal distribution between initial phee and response phee calls in different groups (P1, W10 hand-reared and W10 parent-reared groups). We found that the response phee calls of the parent-reared group at W10 demonstrated an adult-like distribution of response delays (Fig. 6j) [24,30]. The ICI between the initial phee calls and the response phee calls greatly increased from P1, W10 in hand-reared animals to W10 in parent-reared animals (Fig. 6j). In addition, the overlapped phee calls were significantly decreased from P1 to W10 in hand-reared or from P1 to W10 in parent-reared marmosets (Fig. 6k, one-way ANOVA, F = 15.236, *P* < 0.001). However, the proportion of overlapped phee calls between W10 hand-reared and W10 parent-reared marmosets was not statistically significant (Fig. 6k, one-way ANOVA followed by Bonferroni's test for multiple comparisons, *P* = 0.250). We also analysed the temporal distribution between the initial phee to all types of response calls in the different animal groups (Fig. S5i). We found that the hand-reared group at W10 showed a similar cumulative curve to that of the P1 animals (Fig. S5i, right panel). In addition, the proportion of overlapped calls in the hand-reared group at W10 was not different from that of the P1 animals but was significantly lower than that in parent-reared animals at W10 (Fig. S5j, one-way ANOVA, F = 8.092, *P* < 0.005). So, the parent-reared experience may influence the development of vocal communications, resulting in the differences in the gap of silence as well as the proportion of overlapped calls between phee-phee and phee-other call pairs. Taken together, these results further indicated that turn-taking in vocal interactions of infant marmosets is improved by the postnatal phonetic environment.

## DISCUSSION

Although human speech and animal vocalizations are crucial for social communication, their developmental mechanisms remain unclear. Here, by systematically recording the vocalizations of infant marmosets in an antiphonal calling scenario, we studied the ontogeny of vocal turn-taking of marmosets that were raised under different postnatal phonetic environments. Our results showed that P1 marmosets emitted not only immature calls, but also various types of adult-used calls (Fig. 1). These calls provided the acoustic structural basis for vocal communication, which was further proved by the presence of vocal antiphony on P1 (Figs 2 and 3). Moreover, we found that the postnatal sound environment during early development affected not only the emitted call types and spectrotemporal structures of calls, but also the call transitions and turn-taking behaviors during social interaction in an antiphonal calling scenario (Figs 4–6). These results provide direct evidence that the ability for turn-taking in marmoset vocal communication exists but is premature at birth, while it can be refined by parental vocal interactions and social feedback during early development.

### Although the vocal production is predetermined, the acoustic parameters are premature at birth

The babbling of human infants is an important vocal developmental stage before language acquisition; it undergoes dramatic changes and becomes more speech-like in the first postnatal year [6,10,19,20]. The vocal development of human infants is a learning process in which infants imitate the speech of caretakers through vocal interactions [20]. However, the vocalizations of non-human primates, including marmosets, are widely believed to be innate and undergo little or no production-related acoustic changes during development [1,6,15,16]. The direct evidence is that infants who are born deaf or those who are raised in sound-isolated environments could produce all repertoire of conspecific calls [15,16]. However, recent studies have challenged this view, demonstrating that the vocalizations of infant marmosets also undergo dramatic changes and are influenced by parental vocal feedback [4,13,14,32]. Meanwhile, Takahashi *et al.* recorded the marmoset vocalizations on P1 and demonstrated that phee calls in adults were transformed from infant-only calls: cry, phee-cry and subharmonic [4]. Thus, a question was raised about which aspects of vocalization are predetermined and which parts depend on postnatal development. By recording vocalizations of P1 marmosets and during early vocal development, we found that P1 marmosets were able to produce the whole repertoire of the marmoset vocalizations (Fig. 1) and mainly emitted phee calls in an antiphonal calling scenario (Figs 2 and 3). These results confirmed that marmosets’ vocal production is largely predetermined. At least, we cannot exclude the effects of the genetic background on the vocal production of non-human primates [1]. The difference between our findings and Takahashi's results regarding the phee production on P1 may be due to the context difference during vocal recordings, which suggests that marmoset vocalizations are produced in a context-dependent manner, even on P1 [33]. Furthermore, the acoustic parameters, call usage (including the number and percentage of call types), call sequences and the ICI between the initial and response calls dramatically changed with age and the postnatal phonetic environments (Figs 4–6 and Fig. S3), suggesting that the vocal production could be further shaped and refined by the postnatal phonetic environment. In summary, our results demonstrated that, although call types of marmoset vocalizations are largely innate, the basic acoustic structures and context-dependent call usage are shaped by the postnatal phonetic environment during early development.

### Vocal antiphony in marmosets is present at birth

Turn-taking is a fundamental feature of human conversation; it requires two speakers to precisely coordinate the timing of syllables and sentences while talking [12,17]. Human turn-taking during conversation is a learned skill during early vocal development via social interactions with caretakers [30,39]. The *Callithrichids*, including marmosets, are one of the few non-human primate species that exhibit monogamous behavior and the couples engage in cooperatively taking care of their young [5,23,40,41]. During care-taking, social interactions, including parent vocal communications, are essential for the vocal development of infants [13,14,32]. In addition, several studies have shown that certain non-human primates, including marmosets, exhibit speech-like antiphonal behavior during vocal interactions with conspecifics [23–25,39]. Takahashi *et al.* recorded the calls between infant and parent marmosets in an antiphonal scenario, providing preliminary evidence of turn-taking vocalizations in P1 marmosets [14]. However, the turn-taking behavior in their studies could have been led by adult marmosets and their analysis included all types of calls rather than focusing specifically on phee, which is regarded as the most important call during antiphonal calling. Thus, the ontogeny of turn-taking vocalization remains unclear. After testing the turn-taking of P1 marmosets in the antiphonal calling scenario, we did find that two infant marmosets produced phee calls alternately on P1 (Fig. 2b). Meanwhile, the proportion of antiphonal phee calls was significantly higher than that of the shuffled dataset (Fig. 3j and k). These findings proved our hypothesis that the vocal antiphony in marmosets exists at birth.

However, infant marmosets are usually carried by parents or family members and they do not need particular vocal exchanges in a normal parental care environment. We then wondered what the functional role of vocal turn-taking was for infant marmosets. In the present study, we observed marmosets’ vocal behaviors when infant marmosets were separated from their parents. We found that both infant marmosets and their parents showed increased calling rates during separation (data not shown). In addition, Yano-Nashimoto *et al.* reported that marmoset infants could convey their needs through context-dependent calls and selectively approach familiar caregivers [31]. These results indicated the importance of vocal communication between infant marmosets and their parents. So, early vocal turn-taking behaviors in infant marmosets may be necessary for specific scenarios, such as falling off the body of their parents or signaling their needs to caregivers.

### Postnatal phonetic environment is crucial for the development of marmoset vocal antiphony

It is generally accepted that turn-taking during conversation is a learned skill in humans [18,42,43]. Children from different regions or countries with different dialects and languages display different speech preferences. These results indicate that human language is mainly shaped by the postnatal phonetic environment, especially by family members [18,42,43]. Here, we compared the vocal turn-taking behaviors between parent-reared and hand-reared baby marmosets in which parental and family care was replaced by human maternal care. In addition, although vocal communication exists among infant marmosets, the hand-reared marmosets were never exposed to adult conspecific vocalizations and also lacked vocal interactions with adult marmosets during development. Our results demonstrated that the proportion of phee calls associated with vocal communication in the antiphonal calling scenario did not increase with development in the hand-reared group (Figs 4d and 5). Meanwhile, marmoset babies that were deprived of vocal and social interactions with adult conspecifics during early development displayed a trend for a shorter ICI between initial and response phee calls (phee-phee) or between initial phee and other response call types (phee-other) in the antiphonal calling scenario compared with the parent-reared marmosets (Fig. 6j and Fig. S5). In addition, the calls from parent-reared marmosets only transitioned between phee calls or from phee calls to other call types. However, the calls of hand-reared marmosets at the same age still showed call transitions that were similar to those of P1 marmosets in which their calls switched between any call types (Figs. 4h and Fig. S4). Lastly, we found that the ICI of parent-reared groups increased with development (Fig. S5c) whereas the ICI of the hand-reared group did not (Fig. S5g). These results suggest that a lack of vocal and social interactions with conspecifics, especially with parents, greatly hinders the development and maturation of antiphonal communication. Previous studies have also found that infant marmosets could use the calls to tune to the caregiver's parenting style, such as using negative calls to reject neglectful caregivers, while family-deprived infants fail to develop this kind of call usage [31]. These results echo our results that parental care, especially vocal interaction, is essential for the maturation of call usage. Therefore, our results are consistent with those of previous studies that vocal production, vocal antiphony [30,39], as well as the call usage of marmosets [32] are improved by parental vocal interactions.

### Comparison of vocal ontogeny among humans, non-human primates and other animal species

Vocal ontogeny has been broadly investigated regarding the vocal production and vocal learning in different animal species, including humans [17,18,39], non-human primates [4,7,13,16,19,44], birds [3,20,45], mice [46] and bats [10]. Regarding vocal production, except for humans, most animal species emit almost all repertoires of vocalizations at birth [1,3, 54]. Animals that are born deaf display normal vocal ability even without external acoustic inputs, demonstrating that their vocal production is largely innate [15]. This point of view is also confirmed by the present study in marmosets, which emit most repertoires of adult-like call types at P1 (Fig. 1). On the other hand, only a few species, including humans [6,20], dolphins [47], songbirds [3,20,48] and bats [10], display vocal learning. In humans, vocal development has a critical period in which vocal communication undergoes dramatic changes in acoustic features, produces different words or calls and creates new sequences by using different words to fulfill vocal communication through learning [1,49]. Similarly to humans, animals with the ability of vocal learning display a babbling stage and have a critical period during vocal development [10,20,48]. In addition, these animals can imitate sounds to produce calls during vocal learning [1,5,10,21,45,47,48], which is found by the involvement of the frontal cortex [6]. Non-human primates cannot imitate the vocalizations of other conspecific callers, nor can they learn to produce new types of calls [9]. However, context-related vocal learning was found in previous studies in which non-human primates could be trained to produce calls in response to sensory stimulation under operant conditions [50]. So, non-human primates can flexibly use their calls under special conditions to convey context-related information or their emotions [1,9,26]. Recently, Koda *et al.* compared the motor learning of touching and vocalization in macaque monkeys. They found that vocal learning in macaque monkeys required extended training and reaction time, and the monkeys exhibited difficulty with vocal execution when the visual stimulus appeared earlier than expected [50]. Marmosets have the babbling-like stage during vocal development [19] and vocal turn-taking [14,23], which was thought to be a learned skill during vocal ontogeny [30]. However, we found that the skill of vocal conversation was present at P1(Figs 2 and 3) and could be improved by social interaction with parents during their development (Figs 4 and 6). Thus, the marmoset may serve as an intermediate animal model between other non-human primates and humans for investigating vocal learning.

### Limitations and summary of the current study

The hand-reared condition may have limitations, such as broad influences on marmoset behaviors, especially on social behavior. Although we have strictly controlled experimental conditions, the effect of the postnatal phonetic environment cannot be dissociated from the social environment. So, we cannot exclude the social changes during the early hand-rearing experience that may have modulated vocal production and vocal interactions. Taken together, our results demonstrate that, although vocal production and antiphonal communication in marmosets exist at birth, the basic spectrotemporal acoustic structures and context-dependent call usage including vocal antiphony as well as the call transitions of marmosets are premature and shaped by the postnatal phonetic environment during early development.

## MATERIALS AND METHODS

All experimental procedures were approved by the Animal Use and Care Committee of Zhejiang University and followed the National Institutes of Health guidelines (ZJU20190079).

Detailed materials and methods are available in the Supplementary data.

## Supplementary Material

nwaf162_Supplemental_Files

## Data Availability

Although the data files are large, the original data that support the findings of this study are available upon reasonable request.
