# Supplementary material for: Vocal taking turns is premature at birth and improved by the postnatal phonetic environment in marmosets
Source: Natl Sci Rev. 2025 Apr 24;12(7):nwaf162. doi: 10.1093/nsr/nwaf162 (PMC12239203; doi:10.1093/nsr/nwaf162)
Supplement: nwaf162_Supplemental_Files [file nwaf162_supplemental_files.zip › Qi et al. Supplementary Materials and Methods.docx]

**Supplementary Materials and Methods**

All experimental procedures were approved by the Animal Use and Care Committee of Zhejiang University and followed the National Institutes of Health guidelines.

**Animals**

Sixteen infant common marmosets (*Callithrix jacchus*), bred in the Non-human Primate Center of Zhejiang University, were used in this study. Sixteen newborn marmosets (2 single, 4 twins and 2 triplets) were used to examine the vocal production at postnatal day one (P1), and ten marmosets among them (2 twins and 2 triplets) were used to investigate vocal interactions in the antiphonal calling scenario at P1. Eight marmosets (2 hand-reared twins and 2 parent-reared twins) were used to examine vocal interactions in the antiphonal calling scenario from postnatal week five (W5) to week ten (W10). The body weights of P1 marmosets were in the range of 24.8 to 35.6 g. The marmosets were held in pairs or family groups with enough space and fresh air in rooms with 24-hour ventilation systems, as described in our previous study[1]. The colony room was kept under a 12:12 h day/night cycle with a temperature between 26 and 28°C and 45–55% relative humidity. Food (cereal, eggs, sweet potatoes, honey, fruit, vegetables, and mealworms) and water were freely taken. Additional treats, such as marshmallows, biscuits, or yogurt were used as reinforcements after the transfer of the animals from their home cage to the experimental room.

**Hand-rearing Method**

The hand-rearing method follows our previous study [2]. Some female marmosets (4 females in the present study) give birth to twins and triplets (2 twins and 2 triplets in this study); however, one or two babies may drop off from the parent’s back due to weakness or insufficient milk which will be neglected by parents[3-5]. Furthermore, some parents neglect all their babies due to a lack of nursing experience or other unknown reasons. To save as many babies as possible, we developed a hand-rearing method.

During the hand-rearing stage, each baby marmoset was placed on the body of a stuffed toy monkey in an animal incubator (Autoelex MX-BL600, RCOM, Korea, 855mm*470mm*440mm) with adjustable temperature and humidity. To decrease the stress in the incubator, the stuffed monkeys were placed in the baby’s family cage for at least one day before being put in the incubator. We set the incubator’s temperature at 35°C from P1 to P15. Then, the temperature in the incubator was gradually decreased in 0.5°C steps every 1-3 days until it reached 26°C. The humidity was set at 40%. To avoid social isolation and under animal warfare, hand-rearing was generally performed on two babies simultaneously from twins or triplets. For triplets, two were chosen for hand-rearing and one for parents-rearing. If the newborn baby was found falling off from the parent’s back, it was chosen for hand-rearing. If the triplets can hold their parent's body firmly, two of them were randomly selected for hand-rearing. The food included baby formula (Mead Johnson, USA) and baby rice cereal (Heinz, China). Dietary supplements included probiotics (Puper Intestinal, China). The babies were fed 7 times per day at P1-15 (from 08:00 to 22:00) and this was gradually decreased to 3 times per day at P50 when the infant marmoset was weaned. After 50 days of hand-rearing, the baby marmosets were returned to the colony and raised separately in a small cage (40 cm × 40 cm × 40 cm) next to their family cage for 7 days. Then, they were reunited with their parents and siblings in the home cage at the age of ~2 months. To investigate how the postnatal phonetic environment affects the vocal communication of infant marmosets, animals in the hand-reared group (n=4) were reared in a double-walled sound-proof chamber next to the room housing for parent-reared animals. Therefore, animals in the hand-reared group did vocalize and had vocal communications between hand-reared infants, however, they did not hear adult vocalizations, especially from their parents, which provided us with the opportunity to address whether parental vocal feedback affects the development of taking turns in the antiphonal calling scenario.

**Vocal Recordings**

Marmoset vocalizations were recorded in a double-walled sound-proof chamber using condenser microphones (audio-Technica AT2020) placed close to the animals. The sounds were digitized at a sampling frequency of 48 kHz *via* an A/D interface (Roland Rubix 22).

1. **Vocal Recordings from P1 Marmosets**

We recorded the spontaneous vocalizations of P1 marmosets (16 marmosets, recorded individually) for ~5 min. They were usually carried on their parent’s backs. To minimize the stress and human disturbance to the animals, we carried out the experiments in several steps as follows: (1) the baby marmosets and the parent carrying them were lured into a marmoset carrier (32×21×26 cm^3^) using food treats and transferred to the double-walled sound-proof chamber with the same temperature and humidity as the colony; (2) the animals were allowed to adapt to the environment for 10 min; (3) the baby was separated from its parent and wrapped in a warm blanket; (4) the vocal recording was performed for 5 min including 2 sessions. The baby marmoset was separated from its parent and held by the experimenter’s hand where the infants couldn’t visually contact with their parents and the calls were recorded for 2 minutes(1^st^ session); then, we placed the baby in front of the marmoset carrier where the infants could visually contact with their parents and recorded for another 3 minutes (2^nd^ session). and (5) the babies were either returned to the colony with their parents or received hand-rearing in the animal incubator in the double-walled sound-proof chamber.

**(b) Vocal Recording from P1 Marmosets** **in the Antiphonal Calling Scenario**

To determine whether the P1 marmosets take turns during vocal communications, we recorded the vocalizations of pairs of P1 sibling marmosets (10 animals, 2 twins and 2 triplets, 8 pairs) in the antiphonal calling scenario for 5-6 min. The animal preparation was the same as in (a). The parents were taken out of the sound-isolated recording room. The two animals were placed at the two ends of the animal incubator inside the sound-proof chamber and kept 25 cm apart by an acoustically transparent but visually opaque curtain (Fig. 2A, upper panel). The calls of the animals were recorded by two microphones placed outside the incubator and next to each animal. According to the intensity difference of each call collected by the two microphones, we could identify the source of each call recorded. After the vocal recording, the infant marmosets were either returned to the colony with their parents or to the animal incubator in the double-walled sound-proof chamber for hand-rearing. Note, only the vocalizations of P1 marmosets (10 marmosets, 2 twins, and 2 triplets) were recorded in the animal incubator.

**(c) Vocal Recordings in the Antiphonal Calling Scenario between W5 and W10**

To determine how the postnatal phonetic environment affects the development of antiphonal calling, we recorded the vocalizations of pairs of baby sibling marmosets (8 animals, 4 hand-reared and 4 parent-reared infants, recorded in pairs) for 10 min twice per week in the morning time from W5 to W10 in the antiphonal calling scenario. In this experiment, we did not record the pairs of one individual hand-reared and one parent-reared. These recording sessions were carried out in the sound-proof chamber for both the hand-reared and parent-reared groups (the pairs came from the same group). The hand-reared animals usually hold the body of a stuffed toy monkey placed in the incubator daily and don’t leave the stuffed toys. So, the stuffed toy monkey and the animal were moved together to a transfer carrier before vocal recordings. Because two groups of animals were housed in two separate rooms next to each other on the same floor, they were taken to the vocal recording room via a similar route, distance, and surrounding environments. Before W5, any disturbance to the animal was minimized except for the P1 vocal recordings. Before a vocal recording session, the animals were taken to the recording room and left for 30 min per day for 7 days to adapt to the environment. Also, during vocal recording, the animals were kept in their familiar transfer carrier to reduce stress and anxiety. We have visually observed their vocal behaviors during transferring and habituation, and both groups displayed similar vocal and motor behaviors (data not shown). The antiphonal calling scenario was set similarly to the condition (b). Thus, the animals were visually occluded but could acoustically interact with each other. Recordings were started after the experimenter left the room and closed the door. Each vocal recording session lasted for 10 min.

**Acoustic Analysis**

The acoustic features of marmoset vocalizations were analyzed using Raven Pro software (Cornell Laboratory of Ornithology, Ithaca, New York). To identify vocalizations from background signals, the acoustic signal was first passed through a 3-18 kHz band-pass filter[6] and the averaged amplitude of silent periods without marmoset calls was selected as the baseline. To identify the individual calls, the spectrogram of segmented calls was plotted using a fast Fourier transform (1024 points, Hanning window, and 50% overlap) with Raven Pro software. A single call in the spectrogram was selected using a rectangle box, and then spectrotemporal information was measured using the Raven Pro software. The different types of calls can be spatially (frequency) and temporally (time) separated in the sonogram figured with Raven Pro software. The onset of each call was determined as the beginning time of amplitude significantly higher than the baseline. The offset of each call is determined as the end time of amplitude begins to be less than the baseline. The onset and offset of each call were also double-checked manually. An inter-call interval (ICI) was the time between the offset and onset of two consecutive calls. An inter-onset interval (IOI) was defined as the time interval between the onset of two consecutive calls. The fundamental frequency (f0), duration, center frequency, and entropy in each segmented call were measured by Raven Pro software. f0 is the lowest frequency component among the harmonic frequency components of a call. We also compared the mean, median, begin, end, minimal, and maximum frequency of f0 in different types of calls between “within-individual” and “between-individuals” calls as well as between parent-reared and hand-reared groups. Call duration was calculated as the time difference between the onset and the offset of a call. The center frequency was defined as the frequency with the maximal energy within a call. The dominant frequency is the frequency corresponding to the maximum in the spectrum[7]. Wiener entropy was used to measure the power spectrum of a specific call and was calculated as the logarithm ratio between the geometric and arithmetic means of the power spectrum[7]. A high entropy corresponds to the broadband spectrogram of a call, whereas a pure tone would have zero entropy. Last, different call features were compared in different groups.

The calls in the antiphonal calling scenario were recorded by two microphones. To identify the caller of a call, we first adjusted the waveform of the whole recording session (5 minutes in P1 animals and 10 minutes for W5 to W10 animals) from two microphones in the same amplitude range; then we plotted the spectrograms of calls from two microphones; last, the spectrograms of calls from two microphones were subtracted. Then, we obtained the difference of spectrograms between the two microphones. Only the caller produced a call with an intensity difference between two microphones higher than 5 dBFS (>5 dBFS) was identified and used, and others as unidentified caller and call were not used for analysis. If the sonograms of one call are positive and higher than 5dBFS in the differential spectrograms, the call is assigned to the caller close to the subtractor microphone, or the call is assigned to the caller close to the minuend microphone. For the time-overlapped calls, most were separated by the frequency differences in the y-axis in the spectrogram. The calls with spectrum overlapped could also be selected using a rectangle box, and then the spectrotemporal information is measured using the Raven Pro software (Fig. S1 A-D). Both time and frequency overlapped calls in two marmosets were not used because of the difficulty in measuring spectrotemporal information (Fig. S1 C-D). Totally, ~90% of calls could be identified (Fig. S1), and ~85% of phee calls could be identified and separated in an antiphonal scenario (Fig. S2B).

The probability (vocal production) is the ratio between the number of animals that make a particular call and the total number of animals. The calculation is

$$Probalibility=\frac{nC}{n}*100\%$$

n is the animal number, and C is the number of animals displayed in a particular call. For example, all P1 baby monkeys produced phee, so the probability of phee is 100%.

**Identification of Different Call Types**

The segmented vocalizations recorded from infant marmosets were first manually classified through visual inspection based on the spectrotemporal patterns of the calls. Based on previous studies[3, 6, 8], 9 types of calls (phee, twitter, trill, trill-phee, cry, compound-cry, sub-harmonics, tsik, and ekk) were identified manually. These calls have distinct spectrotemporal profiles [3, 6-8]. Phee is defined as a faint, pure tone-like high-pitch call with a duration between 0.11-0.45 s and with a center frequency between 7-10 kHz. Generally, Phee begins with a short upward frequency-modulated (FM) sweep those transitions to a long flat or gradually ascending FM sweep and has a larger variability at the end[6]. A twitter is a sequence of short (0.05-0.09 s), high-pitched notes uttered at intervals of 0.08-0.11 s. The number of notes in a twitter varies from 2 to 5 in babies[9, 10]. Trill is distinguished from phee by its characteristic sinusoid-like FM structure throughout the call with relatively low intensity. Like Phee, a trill’s fundamental frequency is narrowband and is typically uttered between 5-8 kHz [3, 6-8]. Trill-phee is an intermediate call type between phee and trill, which is identified as beginning with a sinusoidal FM segment that dampens into a slowly rising linear FM segment. The intensity of trillphee is between phee and trill [3, 6-8]. Cry is a broadband call with a fundamental frequency of ~ 3.5 kHz; the compound-cry is a combination of a cry and another call, regardless of the order. Sub-harmonics is subharmonic phees[10]. Tsik is a short broadband call consisting of a linearly ascending FM sweep that merges into a sharply descending linear FM sweep[10]. Ekk is a short call that is defined as one of the lowest-frequency calls[3]. After visually recognizing the calls, we calculated the mean and standard deviation of major acoustic parameters of these visually identified call types. Then, we fitted these major acoustic parameters into a normal distribution and used the 95th percentile as the threshold to define the different types of calls. Visually selected phee calls that outside of the 95% percentiles of major acoustic parameters were classified as ‘phee-like’ calls. Similarly, Trill, Trillphee, Twitter, Cry, and Tsik are further defined by key acoustic parameters.

**The Analysis of “within-individual”** **Calls and “between-individuals” Call****s**

First, we identified the caller based on the spectrogram and amplitude of each call collected by the two microphones. Then, each call was assigned to one of two categories based on the caller of the preceding call: either a “within-individual” call or a “between-individuals” call[11]. A call was defined as a “within-individual” call if the preceding call was emitted by the same individual or a “between-individuals” call if the preceding call was from another caller (Fig. 2A. bottom panel). Then, we compared the acoustic parameters of “between-individuals” and “within-individual” calls, including the inter-ICI, IOI, center frequency, Wiener entropy, f0 Begin, f0 End, f0 Min f0 Median, f0 Max, and f0 Mean using Two-way ANOVA followed by Bonferroni’s test.

To decrease the individual variability, we merged different acoustic parameters of the same individual in different postnatal weeks together and then normalized them using the z score. Last, we compared the difference between “within-individual” and “between-individuals” calls for P1, and postnatal W5-W10 of parent-reared and hand-reared groups after the calls were normalized using Two-way ANOVA followed by Bonferroni’s test.

**The Analysis of Initial Call and Responded Call.**

We separated each “between-individuals” call pair into 'initial call' and 'response call' based on the temporal sequence of calls from the two callers. Then, we compared the acoustic parameters, including ICI, IOI, center frequency, Wiener entropy, f0 Begin, f0 End, f0 Min f0 Median, f0 Max, and f0 Mean between 'initial call' and 'response call' using Two-way ANOVA followed by Bonferroni’s test.

**Identification of Antiphonal Calling**

The gap of silence between the initial and response calls (ICI) was defined as the time between the offset of the initial call and the onset of the response call; this provided the basis for analyses of vocal interactions between two animals in subsequent statistical calculations. Previous studies used a 5 s window (mean+ standard error) as the ICI threshold (< 5s) to define antiphonal calls in adult animals[11, 12]. Because newborn marmosets usually produce calls at short ICI, we calculated the ICI of all recorded calls. The ICI threshold for antiphonal calls was recalculated using the same method in adult animals (mean + std)[11]. In addition, antiphonal communication had to meet the following criteria: (1) vocal exchanges between two animals; (2) using the same type of call during vocal exchanges; and (3) the ICI is less than the threshold [11, 13]. This criterion has been used in all antiphonal calling analyses. The proportion of antiphonal calling was also measured in the shuffled dataset and compared with the experiment dataset.

**The Call Shuffling Methods**

The call shuffling in the antiphonal scenario was performed using a custom-made MATLAB script. To shuffle the calls, we first identified the time of the calls (locations), call types, and the callers of each call in each recording session; the calls were tagged in an increased time sequence (1,2 …number of calls). Then, we randomized the call tag and rearranged calls into a new sequence, including the time, types, and caller information. Last, we inserted the calls of the new sequence into the original sound signal. Then, we used the same criterion, including ICI, call types, and alternated call or not, to identify the proportion of antiphonal calls in the shuffling dataset. The sound signal was shuffled 1000 times, and the proportion of antiphonal calls in the shuffling dataset was averaged. We compared the proportion of antiphonal calls between the experimental dataset with the shuffling dataset using Two-way ANOVA followed by Bonferroni’s test.

**Normalized Probability Plots**

To characterize the ICI between the initial phee and all response calls produced by the two animals, we chose initial phee and response call pairs and analyzed the ICI distribution between initial phee to response call pairs. The histograms were time-locked to the offset of the initial phee call (0 on the x-axis). The time bins were 0.1 s. The distribution in each histogram was normalized by the number of call pairs. Thus, the summation of the area under the normalized histogram curve would be 1. We also analyzed the ICI distribution between the initial phee to response phee which was normalized by the number of phee-phee call pairs.

**Cross-correlation Analysis of Vocal Communication**

The cross-correlation coefficients among the calls from two baby marmosets were calculated based on previous studies[13, 14]. To characterize the reciprocity in vocal communication between two baby marmosets, we analyzed the cross-correlation coefficients of the calling events from two animals in the antiphonal calling scenario. In detail, a call sequence was digitized into a sequence of “1” and “0” in discrete time bins 0.1 s. The position of “1” was used to indicate the calling period and “0” was the no-call period. The cross-correlation coefficients between the calls from two animals were calculated using the code of ‘xcorr' provided by MATLAB. The curve of cross-correlation coefficients was normalized by subtracting the average value. We also separately calculated the cross-correlation coefficients of the between-individual or within-individual calls using similar methods.

**Transition Diagrams of Vocal Sequences**

The transition diagrams were calculated using previous methods[3]. Here, only the calls with ICI less than the threshold of antiphonal calling were used. We included the overlapped calls in this analysis and treated them as normal calls. One step of transition diagrams was calculated from the vocal sequences of 10 P1 marmosets, 4 hand-reared and 4 parent-reared marmosets at the age of W5 and W10. We also plotted the transition diagrams of vocal sequences between “within-individual” and “between-individuals” for different groups.

**Clustering Dendrogram**

Based on call distribution and transitions, the clustering dendrogram was calculated from 4 hand-reared and 4 parent-reared marmosets at the age of W5 and W10. We used the 'cluster' algorithm, an agglomerative hierarchical cluster tree provided by Matlab. This algorithm merges the two clusters with the smallest distance by calculating the distance between clusters until there is only one cluster.

**Display the Temporal Relationship between the Initial Phee Call and All Response Calls**

To display the time relationship between the initial and response calls, we calculated the duration, ICI and IOI between initial and response phee calls. Then, the initial phee calls and the response calls were aligned by the offset of the initial phee calls and sorted by the length of ICI for each type of responded call (Fig. 6 A-C).

**Statistical Analyses**

All values are expressed as the mean ± SEM (standard error of the mean). The Kolmogorov-Smirnov test was used to test whether two cumulative distribution functions are different. Statistical differences between the two groups across age were compared using Two-way or One-way analysis of variance (ANOVA) followed by Bonferroni's test for multiple comparisons **(List in Supplementary Table 1-7)**. The student-paired t-test was used to compare the two groups at the same age if the data were normally distributed. Otherwise, the Wilcoxon or Mann-Whitney test was used. P values with P <0.05 were considered statistically significant in the present study. * P<0.05; **P<0.01; ***P<0.001

1. Cao X, Zhu L, Qi R *et al.* Effect of a High Estrogen Level in Early Pregnancy on the Development and Behavior of Marmoset Offspring. *ACS Omega*. 2022; **7**(41): 36175-36183. doi: 10.1021/acsomega.2c03263

2. Sun H, Li R, Lin Y *et al.* Hand-Rearing Method for Infant Marmosets. *J Vis Exp*. 2023(196). doi: 10.3791/65296

3. Gultekin YB, Hage SR. Limiting parental feedback disrupts vocal development in marmoset monkeys. *Nat Commun*. 2017; **8**: 14046. doi: 10.1038/ncomms14046

4. Landman R, Sharma J, Hyman JB *et al.* Close-range vocal interaction in the common marmoset (Callithrix jacchus). *PLoS One*. 2020; **15**(4): e0227392. doi: 10.1371/journal.pone.0227392

5. Poole TB, Evans RG. Reproduction, infant survival and productivity of a colony of common marmosets (Callithrix jacchus jacchus). *Lab Anim*. 1982; **16**(1): 88-97. doi: 10.1258/002367782780908760

6. Takahashi DY, Fenley AR, Teramoto Y *et al.* LANGUAGE DEVELOPMENT. The developmental dynamics of marmoset monkey vocal production. *Science*. 2015; **349**(6249): 734-738. doi: 10.1126/science.aab1058

7. Agamaite JA, Chang CJ, Osmanski MS, Wang X. A quantitative acoustic analysis of the vocal repertoire of the common marmoset (Callithrix jacchus). *J Acoust Soc Am*. 2015; **138**(5): 2906-2928. doi: 10.1121/1.4934268

8. Pistorio AL, Vintch B, Wang X. Acoustic analysis of vocal development in a New World primate, the common marmoset (Callithrix jacchus). *J Acoust Soc Am*. 2006; **120**(3): 1655-1670. doi: 10.1121/1.2225899

9. Epple G. Comparative studies on vocalization in marmoset monkeys (Hapalidae). *Folia Primatol (Basel)*. 1968; **8**(1): 1-40. doi: 10.1159/000155129

10. Gultekin YB, Hage SR. Limiting parental interaction during vocal development affects acoustic call structure in marmoset monkeys. *Sci Adv*. 2018; **4**(4): eaar4012. doi: 10.1126/sciadv.aar4012

11. Miller CT, Wang X. Sensory-motor interactions modulate a primate vocal behavior: antiphonal calling in common marmosets. *J Comp Physiol A Neuroethol Sens Neural Behav Physiol*. 2006; **192**(1): 27-38. doi: 10.1007/s00359-005-0043-z

12. Miller CT, Beck K, Meade B, Wang X. Antiphonal call timing in marmosets is behaviorally significant: interactive playback experiments. *J Comp Physiol A Neuroethol Sens Neural Behav Physiol*. 2009; **195**(8): 783-789. doi: 10.1007/s00359-009-0456-1

13. Takahashi DY, Narayanan DZ, Ghazanfar AA. Coupled oscillator dynamics of vocal turn-taking in monkeys. *Curr Biol*. 2013; **23**(21): 2162-2168. doi: 10.1016/j.cub.2013.09.005

14. Chow CP, Mitchell JF, Miller CT. Vocal turn-taking in a non-human primate is learned during ontogeny. *Proc Biol Sci*. 2015; **282**(1807): 20150069. doi: 10.1098/rspb.2015.0069
