# Supplementary material for: Vocal taking turns is premature at birth and improved by the postnatal phonetic environment in marmosets
Source: Natl Sci Rev. 2025 Apr 24;12(7):nwaf162. doi: 10.1093/nsr/nwaf162 (PMC12239203; doi:10.1093/nsr/nwaf162)
Supplement: nwaf162_Supplemental_Files [file nwaf162_supplemental_files.zip › Qi et al. Supplementary Tables.docx]

**Supplementary tables 1-7**

**Table 1. Statistics of One-Way or two-way analysis of variance**

| Figures | Statistical Methods | Main factor 1 | Main factor 2 | F and p for ANOVA | |
| --- | --- | --- | --- | --- | --- |
|  |  |  |  | **F value** | **p value** |
| Fig.2 d | Two ANOVA, Bonferr | Call types | Between & Within-individuals | 50.046 | <0.001 |
| Fig.2 e | Two ANOVA, Bonferr | Call types | Between & Within-individuals | 12.426 | <0.001 |
| Fig.2 f | Two ANOVA, Bonferr | Call types | Between & Within-individuals | 13.643 | <0.001 |
| Fig.2 g | Two ANOVA, Bonferr | Call types | Between & Within-individuals | 0.596 | 0.551 |
| Fig.2 h | Two ANOVA, Bonferr | Call types | Between & Within-individuals | 6.866 | 0.001 |
| Fig.2 i | Two ANOVA, Bonferr | Call types | Between & Within-individuals | 1.152 | 0.316 |
| Fig.2 j | Two ANOVA, Bonferr | Call types | Between & Within-individuals | 0.071 | 0.932 |
| Fig.2 k | Two ANOVA, Bonferr | Call types | Between & Within-individuals | 1.031 | 0.357 |
| Fig.2 l | Two ANOVA, Bonferr | Call types | Between & Within-individuals | 0.299 | 0.741 |
| Fig.3 a | Two ANOVA, Bonferr | Call types | Initial & Response calls | 1.421 | 0.234 |
| Fig.3 b | Two ANOVA, Bonferr | Initial & Response calls | Call types | 2.206 | 0.027 |
| Fig.3 c | Two ANOVA, Bonferr | Call types | Initial & Response calls | 135.458 | <0.001 |
| Fig.3 d | Two ANOVA, Bonferr | Call types | Initial & Response calls | 0.395 | 0.674 |
| Fig.3 e | Two ANOVA, Bonferr | Call types | Initial & Response calls | 0.644 | 0.526 |
| Fig.3 f | Two ANOVA, Bonferr | Call types | Initial & Response calls | 0.767 | 0.465 |
| Fig.3 g | Two ANOVA, Bonferr | Call types | Initial & Response calls | 1.087 | 0.338 |
| Fig.3 h | Two ANOVA, Bonferr | Call types | Initial & Response calls | 0.091 | 0.913 |
| Fig.3 i | Two ANOVA, Bonferr | Call types | Initial & Response calls | 0.936 | 0.393 |
| Fig.3 j | Two ANOVA, Bonferr | Call types | Experimental & shuffled | 13.290 | <0.001 |
| Fig.3 k | One ANOVA, Bonferr | Call types | na | 26.010 | <0.001 |
| Fig.3 l | Two ANOVA, Bonferr | Initial & Response calls | Overlap & non-overlap calls | 4.808 | 0.029 |
| Fig.4 a | Two ANOVA, Bonferr | Postnatal weeks | Parent & Hand-reared groups | 3.287 | 0.005 |
| Fig.4 b | Two ANOVA, Bonferr | Postnatal weeks | Parent & Hand-reared groups | 7.613 | <0.001 |
| Fig.4 c | Two ANOVA, Bonferr | Postnatal weeks | Call types | 1.600 | 0.227 |
| Fig.4 d | Two ANOVA, Bonferr | Postnatal weeks | Call types | 2.503 | 0.042 |
| Fig.4 e | Two ANOVA, Bonferr | Call types | Parent & Hand-reared groups | 101.65 | <0.001 |
| Fig.5 a | Two ANOVA, Bonferr | Initial & Response calls | Parent & Hand-reared groups | 0.116 | 0.734 |
| Fig.5 b | Two ANOVA, Bonferr | Initial & Response calls | Parent & Hand-reared groups | 0.013 | 0.911 |
| Fig.5 c | Two ANOVA, Bonferr | Initial & Response calls | Parent & Hand-reared groups | 0.838 | 0.362 |
| Fig.5 d | Two ANOVA, Bonferr | Parent & Hand-reared groups | Experimental & shuffled | 0.658 | 0.421 |
| Fig.5 e | Two ANOVA, Bonferr | Parent & Hand-reared groups | Experimental & shuffled | 0.003 | 0.953 |
| Fig.5 f | Two ANOVA, Bonferr | Parent & Hand-reared groups | Experimental & shuffled | 0.017 | 0.898 |
| Fig.5 g | Two ANOVA, Bonferr | Call types | Parent & Hand-reared groups | 1.415 | 0.25 |
| Fig.6 e | One ANOVA, Bonferr | Call types | na | 38.394 | 0.001 |
| Fig.6 f | Two ANOVA, Bonferr | Initial & Response calls | Overlap & non-overlap calls | 8.416 | 0.004 |
| Fig.6 h | One ANOVA, Bonferr | Call types | na | 3.153 | 0.092 |
| Fig.6 i | Two ANOVA, Bonferr | Initial & Response calls | Overlap & non-overlap calls | 0.996 | 0.319 |
| Fig.6 k | One ANOVA, Bonferr | Postnatal Ages | na | 15.236 | <0.001 |
| Fig. S2g | Two ANOVA, Bonferr | Call types | Between & Within-individuals | 12.691 | <0.001 |
| Fig. S2h | Two ANOVA, Bonferr | Call types | Between & Within-individuals | 11.123 | <0.001 |
| Fig. S2i | Two ANOVA, Bonferr | Call types | Between & Within-individuals | 17.685 | <0.001 |
| Fig. S3a | Two ANOVA, Bonferr | Postnatal weeks | Parent & Hand-reared groups | 2.261 | 0.257 |
| Fig. S3b | Two ANOVA, Bonferr | Postnatal weeks | Parent & Hand-reared groups | 10.342 | <0.001 |
| Fig. S3c | Two ANOVA, Bonferr | Postnatal weeks | Parent & Hand-reared groups | 7.178 | <0.001 |
| Fig. S3d | Two ANOVA, Bonferr | Postnatal weeks | Parent & Hand-reared groups | 1.351 | 0.284 |
| Fig. S3e | Two ANOVA, Bonferr | Postnatal weeks | Parent & Hand-reared groups | 2.276 | 0.057 |
| Fig. S3f | Two ANOVA, Bonferr | Postnatal weeks | Between & Within-individuals | 2.174 | 0.041 |
| Fig. S3g | Two ANOVA, Bonferr | Postnatal weeks | Between & Within-individuals | 9.366 | <0.001 |
| Fig. S3h | Two ANOVA, Bonferr | Postnatal weeks | Between & Within-individuals | 7.732 | <0.001 |
| Fig. S3i | Two ANOVA, Bonferr | Postnatal weeks | Parent & Hand-reared groups | 1.565 | 0.114 |
| Fig. S3j | Two ANOVA, Bonferr | Postnatal weeks | Parent & Hand-reared groups | 1.431 | 0.209 |
| Fig. S3k | Two ANOVA, Bonferr | Postnatal weeks | Parent & Hand-reared groups | 6.882 | <0.001 |
| Fig. S3l | Two ANOVA, Bonferr | Different F0 parameters | Parent & Hand-reared groups | 25.568 | <0.001 |
| Fig. S3m | Two ANOVA, Bonferr | Different F0 parameters | Parent & Hand-reared groups | 85.741 | <0.001 |
| Fig. S5c | Two ANOVA, Bonferr | Postnatal weeks | Between & Within-individuals | 62.027 | <0.001 |
| Fig. S5d | Two ANOVA, Bonferr | Postnatal weeks | Between & Within-individuals | 20.062 | <0.001 |
| Fig. S5g | Two ANOVA, Bonferr | Postnatal weeks | Between & Within-individuals | 0.083 | 0.773 |
| Fig. S5h | Two ANOVA, Bonferr | Postnatal weeks | Between & Within-individuals | 0.514 | 0.473 |
| Fig. S5j | One ANOVA, Bonferr | Postnatal Ages | na | 8.092 | 0.005 |

Footnotes: Two ANOVA, Bonferr: Two-way analysis of variance (ANOVA) followed by Bonferroni's test; One ANOVA, Bonferr: One-way analysis of variance (ANOVA) followed by Bonferroni's test; *, p<0.05, **P<0.01; ***P<0.001, ****, <0.001.

**Table 2. Statistics of dataset which only has two groups**

| Figures | Statistical Methods | P value |
| --- | --- | --- |
| Fig.1 e | Wilcoxon test | 0.003 |
| Fig.4 f | Wilcoxon test | 0.001 |
| Fig.4 g | Wilcoxon test | <0.001 |
| Fig. S1e | Paired test | <0.001 |
| Fig. S2b | Paired test | <0.001 |
| Fig. S2e | Mann-Whitney U | <0.001 |
| Fig. S2f | Mann-Whitney U | <0.001 |

*, p<0.05, **P<0.01; ***P<0.001, ****, <0.001

**Table 3. the p values for Figure 2g - 2l**

| Figures | Phee | Twitter | Trill |
| --- | --- | --- | --- |
| Fig2g | <0.001 | .046 | .242 |
| Fig2h | <0.001 | .231 | <0.001 |
| Fig2i | <0.001 | .406 | .004 |
| Fig2j | <0.001 | .317 | .050 |
| Fig2k | <0.001 | .165 | .062 |
| Fig2l | <0.001 | .179 | .015 |

**Table 4. the p values for Figure 3b**

| Initial call | Phee-Twitter | Phee-Trill | Phee-Cry | Twitter-Trill | Twitter-Cry | Trill-Cry |
| --- | --- | --- | --- | --- | --- | --- |
| Phee | <0.001 | <0.001 | <0.001 | 1.000 | 1.000 | 1.000 |
| Twitter | .001 | .013 | .125 | 1.000 | .873 | 1.000 |
| Trill | .015 | .060 | .047 | 1.000 | 1.000 | 1.000 |
| Cry | <0.001 | <0.001 | <0.001 | 1.000 | 1.000 | 1.000 |

**Table 5. the p values for Figure 3d-3i**

| Figures | Phee | Twitter | Trill |
| --- | --- | --- | --- |
| Fig3d | <0.001 | .109 | .107 |
| Fig3e | .305 | .649 | .208 |
| Fig3f | .011 | .881 | .503 |
| Fig3g | <0.001 | .697 | .208 |
| Fig3h | <0.001 | .096 | .114 |
| Fig3i | <0.001 | .467 | .189 |

**Table 6. the p values for Figure 4c**

| Ages | Phee-Twitter | Phee-Trill | Phee-Cry | Twitter-Trill | Twitter-Cry | Trill-Cry |
| --- | --- | --- | --- | --- | --- | --- |
| W5 | <0.001 | <0.001 | <0.001 | 1.000 | 1.000 | 1.000 |
| W6 | <0.001 | <0.001 | <0.001 | 1.000 | 1.000 | 1.000 |
| W7 | <0.001 | <0.001 | <0.001 | 1.000 | 1.000 | 1.000 |
| W8 | <0.001 | <0.001 | <0.001 | 1.000 | 1.000 | 1.000 |
| W9 | <0.001 | <0.001 | <0.001 | 1.000 | 1.000 | 1.000 |
| W10 | <0.001 | <0.001 | <0.001 | 1.000 | 1.000 | 1.000 |

**Table 7. the p values for Figure 4d**

| Ages | Phee-Twitter | Phee-Trill | Phee-Cry | Twitter-Trill | Twitter-Cry | Trill-Cry |
| --- | --- | --- | --- | --- | --- | --- |
| W5 | 1.000 | 1.000 | .182 | 1.000 | 1.000 | 1.000 |
| W6 | .031 | .094 | .005 | 1.000 | 1.000 | .853 |
| W7 | .007 | .004 | <0.001 | 1.000 | .082 | .148 |
| W8 | <0.001 | <0.001 | <0.001 | 1.000 | .155 | .146 |
| W9 | .106 | .037 | .003 | 1.000 | .450 | 1.000 |
| W10 | .305 | .120 | <0.001 | 1.000 | .016 | .042 |
