# Supplementary material for: Vocal taking turns is premature at birth and improved by the postnatal phonetic environment in marmosets
Source: Natl Sci Rev. 2025 Apr 24;12(7):nwaf162. doi: 10.1093/nsr/nwaf162 (PMC12239203; doi:10.1093/nsr/nwaf162)
Supplement: nwaf162_Supplemental_Files [file nwaf162_supplemental_files.zip › Qi et al_supplementary figure legends.docx]

**Supplementary Figure legends**

**Figure S1. Identifiable and unidentifiable marmosets call in the antiphonal scenario.**

(A-D) Examples showing calls with spectrotemporal overlaps produced by two monkeys. Some of them have clear separation in the spectrum (A-B, yellow arrows), and some have spectral overlaps, which cannot be separated by the Raven Pro software (C-D, red arrows).

(E) Proportion of identifiable and unidentifiable marmoset calls in antiphonal calling scenario (n=8 pairs of animals).

**Figure S2. The ICI between “between-individual” and “within-individuals” calls of P1 marmosets in the antiphonal scenario**

1. Examples showing phee-like calls due to [nontypical](link:nontypical) spectrotemporal acoustic structure.
2. The proportion of phee and phee-like calls recorded in the antiphonal calling scenario.
3. The distribution of ICI of “between-individuals” calls for P1 marmosets in the antiphonal calling scenario. ICI is the time difference between the onset of the response call and the offset of the initial calls. The vertical dashed line: the offset of the initial calls. Different colors represent different individual animals (n=8).
4. The distribution of ICI of “within-individual” calls for P1 marmosets in the antiphonal calling scenario. ICI is the time difference between the onset and offset of two adjacent calls from the same caller.
5. The comparison of ICI between “between-individuals” and “within-individual” calls for P1 marmosets in the antiphonal calling scenario. n=8 pairs.
6. The comparison of IOI between “between-individuals” calls and “within-individual” calls for P1 marmosets in antiphonal calling scenario. n=8 pairs.
7. Averaged z-scored entropy of “between-individuals” and “within-individual” calls of P1 marmosets in antiphonal calling scenario.
8. Averaged z-scored center frequency of “between-individuals” and “within-individual” calls of P1 marmosets in antiphonal calling scenario.
9. Averaged z-scored call duration of “between-individuals” and “within-individual” calls of P1 marmosets in antiphonal calling scenario.
10. Cross-correlation of the call sequences of all calls emitted by two marmosets in the antiphonal calling scenario (n=8 pairs). The black line is the average of 8 datasets; the grey area is the standard deviation. Arrows indicate the peaks.
11. Cross-correlation of the call sequences of “within-individual” calls emitted by two P1 marmosets in the antiphonal calling scenario.
12. Cross-correlation of the call sequences of “between-individuals” calls emitted by two P1 marmosets in the antiphonal calling scenario.
13. Distributions of the ICI of phee-phee pairs for infant marmosets at P1 for experimental dataset and shuffled dataset. Red dashed line: the threshold for antiphonal calls. 0, the offset of the initial phee call.

**Figure S3. Changes in different acoustic parameters during vocal development**

1. Body weight of infant marmosets in the parent-reared (blue, n=4) and hand-reared (orange, n=4) groups from postnatal W5 to W10.

(B-E) Comparison of call types (B), average entropy (C), center frequency (D) and phee duration (E) of the parent-reared (blue, n=4) and hand-reared (orange, n=4) groups from postnatal W5 to W10. The calls were produced in the antiphonal calling scenario.

(F-H) Comparison of z-scored entropy (F), z-scored center frequency(G) and z-scored duration (H) of the phee calls in parent-reared and hand-reared groups from postnatal W5 to W10.

(I-K) Comparison of z-scored entropy (I), z-scored center frequency(J) and z-scored duration (K) of “between-individuals” and “within-individual” phee calls from postnatal W5 to W10.

(L) Comparison of the different f0 parameters of phee calls in the parent-reared and hand-reared groups at W5.

(M) Comparison of different f0 parameters of phee calls in the parent-reared and hand-reared groups at W10. A-M, *P<0.05, **P<0.01, ***P<0.0001.

**Figure S4. The call transition of “between-individuals” calls and “within-individual” calls in different groups of animals at W5 or W10.**

1. The call transition of “between-individuals” calls in the parent-reared W5 (Left) and the hand-reared W5 marmosets (Right).
2. The call transition of “between-individuals” calls in the parent-reared W10 (Left) and the hand-reared W10 marmosets (Right).
3. The call transition of “within-individuals” calls in the parent-reared W5 (Left) and the hand-reared W5 marmosets (Right).
4. The call transition of “within-individuals” calls in the parent-reared W10 (Left) and the hand-reared W10 marmosets (Right). The color of each node corresponds to a type of call, and the arrows correspond to the transitions between call types. The sizes of nodes are the proportion of the call type and the thickness of an arrow represents the transition probability. P, phee (green); TW, twitter (black); TL, trill (orange); TP, trillphee (pink); U, unidentified (grey).

**Figure S5. The changes of inter-call interval (ICI) during development**

(A-B) The ICI distributions of “within-individual” and “between-individuals” phee call at the age of W5 (A) and W10 (B) of the parent-reared group in the antiphonal calling scenario. The distributions were normalized by the number of within-individual phee-phee call pairs or by that of between-individuals phee-phee call pairs respectively.

1. The comparison of ICI between “between-individuals” and “within-individual” phee calls for marmosets in the parent-reared group at W5 and W10. n=4 pairs.
2. The comparison of IOI between “between-individuals” and “within-individual” phee calls for marmosets in the parent-reared group at W5 and W10.

(E-F) The ICI distributions of “between-individuals” phee calls and “within-individual” phee calls at the age of W5 (E) and W10 (F) of the hand-reared marmosets in the antiphonal calling scenario. n=4 pairs. The distributions were normalized by the number of within-individual phee-phee call pairs or by that of between-individuals phee-phee call pairs respectively.

1. The comparison of ICI between “between-individuals” and “within-individual” phee calls for marmosets in the hand-reared group at W5 and W10. ­
2. The comparison of IOI between “between-individuals” and “within-individual” phee calls for marmosets in the hand-reared group at W5 and W10. n=4 pairs.
3. The distributions (left) and Kolmogorov-Smirnov analysis (right) of ICI between initial phee to various response calls types among P1 (blue), parent-reared W10 (green) and hand-reared W10 marmosets (orange). The distributions were normalized by the number of “phee-all” call pairs.

(J) The percentages of overlapped initial phee to various response call types at P1, parent-reared W10 and hand-reared W10 marmosets. *P<0.05, **P<0.01, ***P<0.0001.
