# Supplementary figures and images for: Vocal taking turns is premature at birth and improved by the postnatal phonetic environment in marmosets
Source: Natl Sci Rev. 2025 Apr 24;12(7):nwaf162. doi: 10.1093/nsr/nwaf162 (PMC12239203; doi:10.1093/nsr/nwaf162)

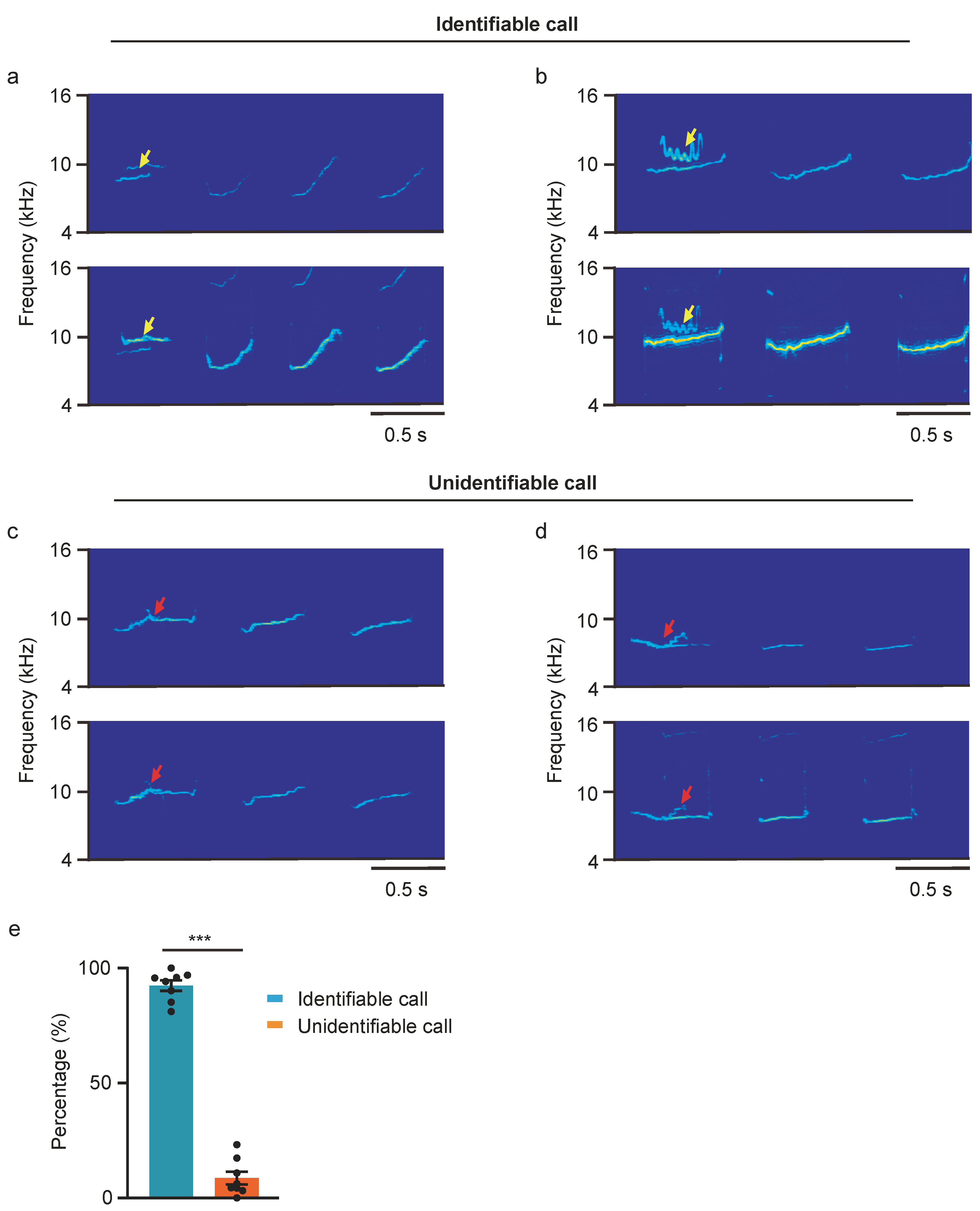

Supplement: nwaf162_Supplemental_Files [file nwaf162_supplemental_files.zip › Qi et al. Supplementary Figures tif/Figure S1.tiff]

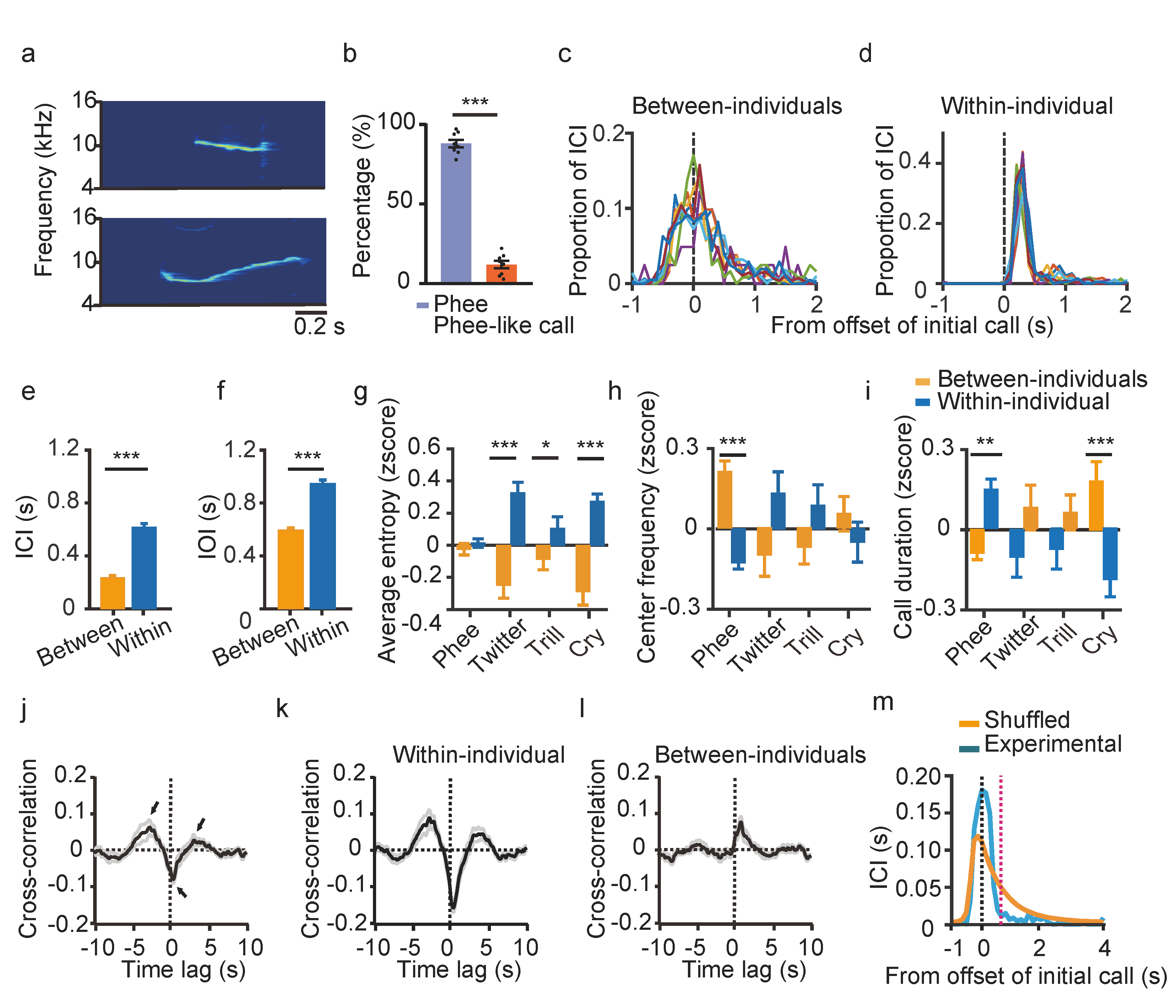

Supplement: nwaf162_Supplemental_Files [file nwaf162_supplemental_files.zip › Qi et al. Supplementary Figures tif/Figure S2.tif]

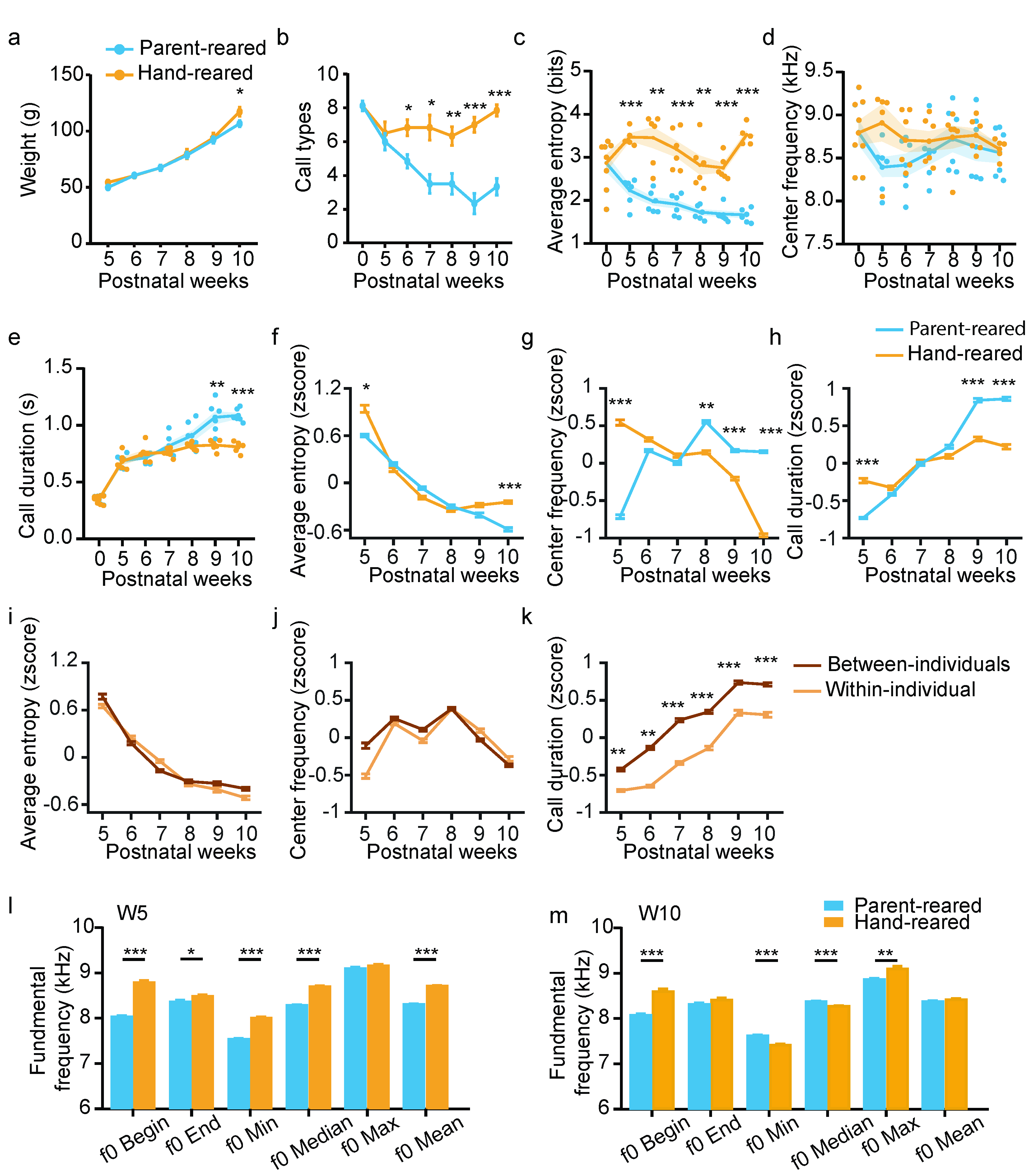

Supplement: nwaf162_Supplemental_Files [file nwaf162_supplemental_files.zip › Qi et al. Supplementary Figures tif/Figure S3 .tif]

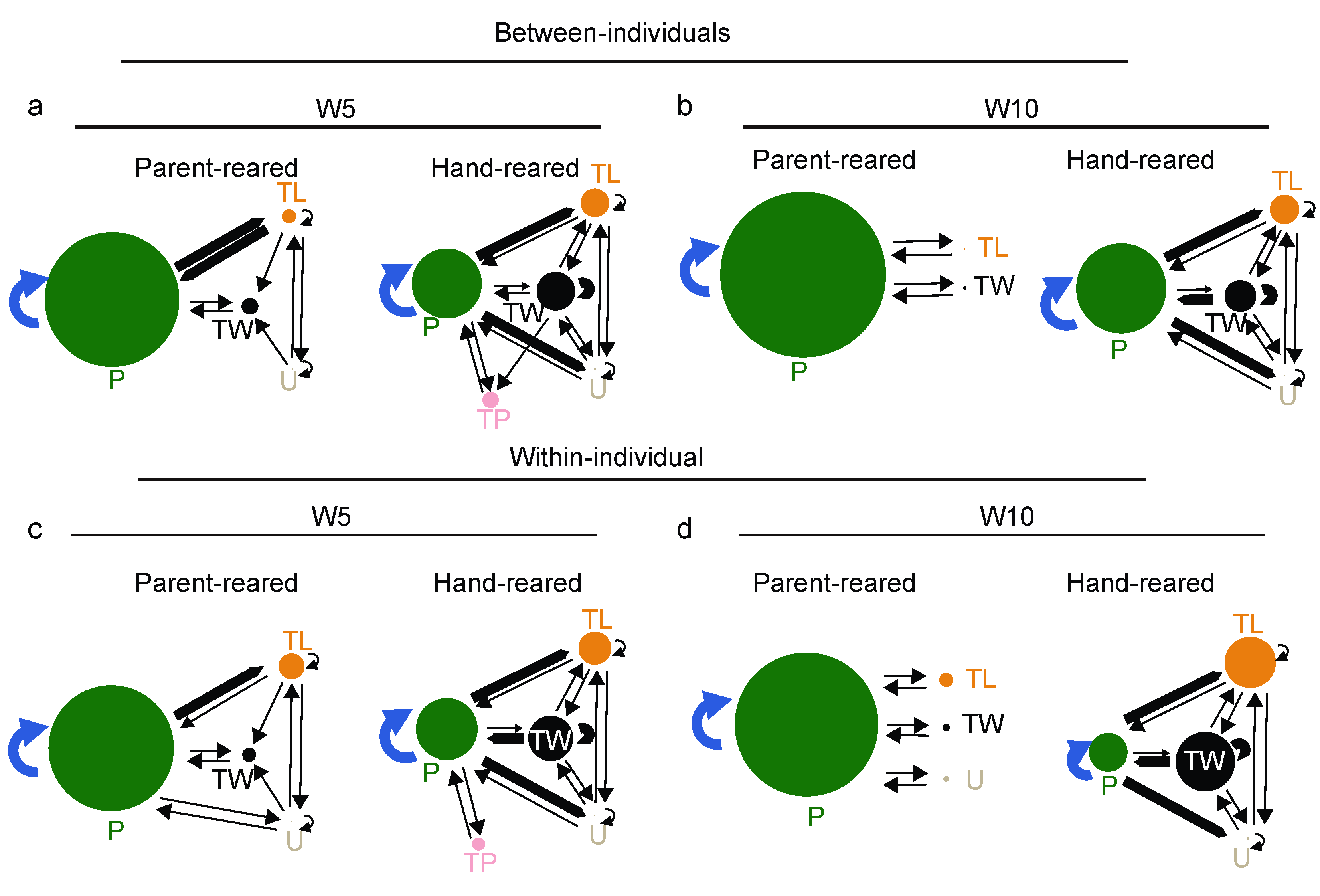

Supplement: nwaf162_Supplemental_Files [file nwaf162_supplemental_files.zip › Qi et al. Supplementary Figures tif/Figure S4.tif]

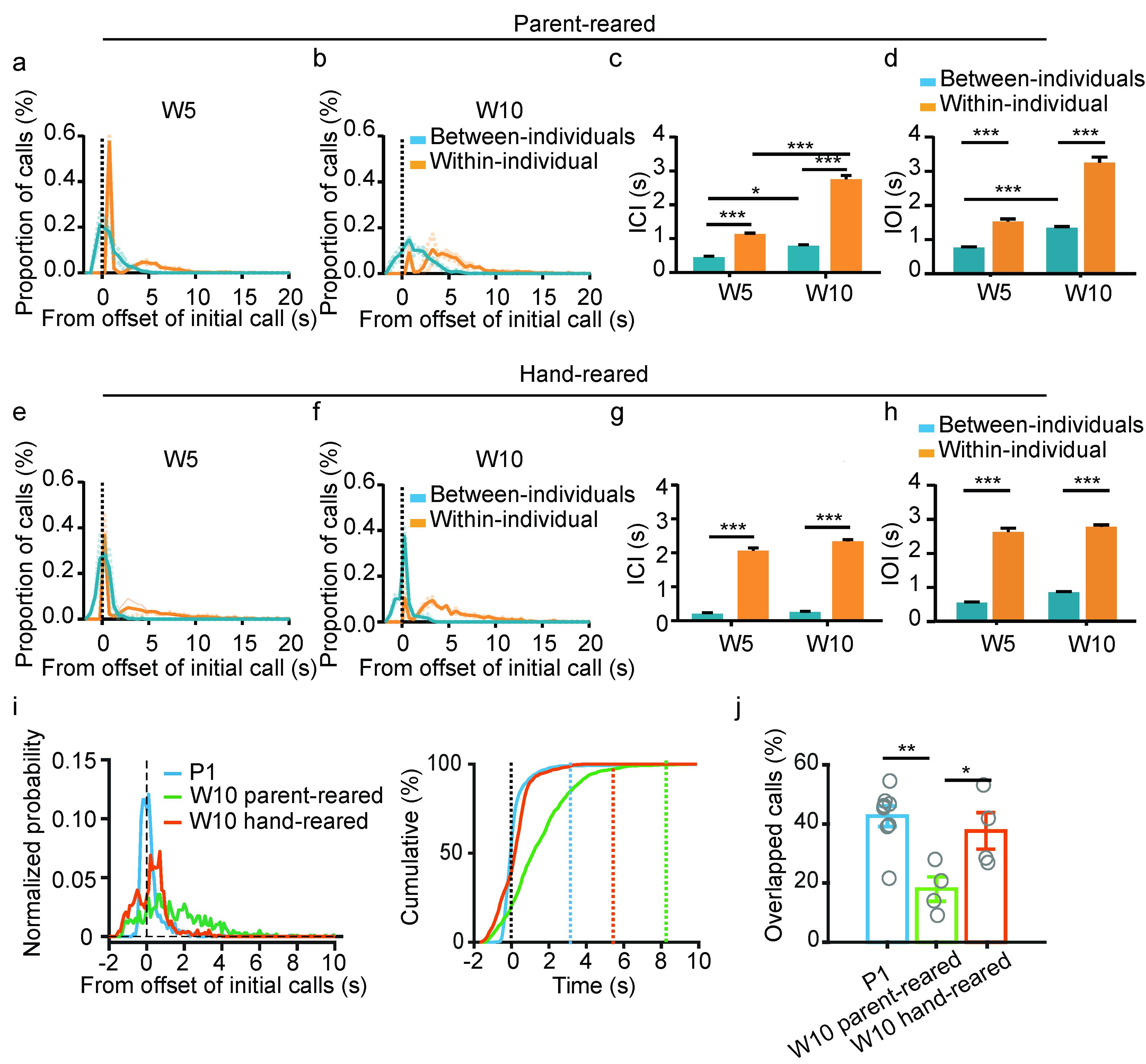

Supplement: nwaf162_Supplemental_Files [file nwaf162_supplemental_files.zip › Qi et al. Supplementary Figures tif/Figure S5.tif]
